# DUF3669, a “domain of unknown function” within ZNF746 and ZNF777, oligomerizes and contributes to transcriptional repression

**DOI:** 10.1186/s12860-019-0243-y

**Published:** 2019-12-19

**Authors:** Mohannad Al Chiblak, Felix Steinbeck, Hans-Jürgen Thiesen, Peter Lorenz

**Affiliations:** Institute of Immunology, Rostock University Medical Center, Schillingallee 70, 18057 Rostock, Germany

**Keywords:** ZNF746, ZNF777, KRAB, ZNF10/KOX1, DUF3669, TRIM28, Parkinson’s disease, HAP1, Transcription, Repression

## Abstract

**Background:**

ZNF746 and ZNF777 belong to a subset of the large Krüppel-associated box (KRAB) zinc finger (ZNF) transcription factor family. They contain, like four other members in human, an additional conserved domain, the “domain of unknown function 3669” (DUF3669). Previous work on members of this subfamily suggested involvement in transcriptional regulation and aberrant ZNF746 overexpression leads to neuronal cell death in Parkinson’s disease.

**Results:**

Here we demonstrate that N-terminal protein segments of the ZNF746a major isoform and ZNF777 act in concert to exert moderate transcriptional repression activities. Full potency depended on the intact configuration consisting of DUF3669, a variant KRAB domain and adjacent sequences. While DUF3669 contributes an intrinsic weak inhibitory activity, the isolated KRAB-AB domains did not repress. Importantly, DUF3669 provides a novel protein-protein interaction interface and mediates direct physical interaction between the members of the subfamily in oligomers. The ZNF746 protein segment encoded by exons 5 and 6 boosted repressor potency, potentially due to the presence of an acceptor lysine for sumoylation at K189. Repressor activity of the potent canonical ZNF10 KRAB domain was not augmented by heterologous transfer of DUF3669, pointing to the importance of context for DUF3669’s impact on transcription. Neither ZNF746a nor ZNF777 protein segments stably associated with TRIM28 within cells. Isoform ZNF746b that contains, unlike the major isoform, a full-length KRAB-A subdomain, displayed substantially increased repressor potency. This increase is due to canonical mechanisms known for KRAB domains since it did not take place in HAP1 knockout models of TRIM28 and SETDB1. A glycine to glutamic acid replacement that complies with a bona fide conserved “MLE” sequence within KRAB-A led to a further strong gain in repressor potency to levels comparable to those of the canonical ZNF10 KRAB domain. Each gain of repressive activity was accompanied by an enhanced interaction with TRIM28 protein.

**Conclusion:**

DUF3669 adds a protein-protein interaction surface to a subgroup of KRAB-ZNF proteins within an N-terminal configuration with variant KRAB and adjacent sequences likely regulated by sumoylation. DUF3669 contributes to transcriptional repression strength and its homo- and hetero-oligomerization characteristics probably extended the regulatory repertoire of KRAB-ZNF transcription factors during amniote evolution.

## Background

C2H2/Krüppel-type zinc finger (ZNF) proteins with Krüppel-associated box (KRAB) domain form the largest family of transcription factors in human with more than 400 members [1, 2]. While the functions of most of them await to be investigated, individual members have been demonstrated to participate in biological processes such as transcriptional gene regulation, differentiation, development, imprinting and in the restriction of endogenous retroelements [3–5]. The KRAB domain was first described as an N-terminal heptad repeat of leucines in the ZNF protein ZNF10/KOX1 [6]. Typical KRAB-ZNF proteins are composed of an amino-terminal KRAB domain and a carboxy-terminal array of multiple zinc fingers [6, 7]. The KRAB domain itself is made up of canonical subdomain-A with or without an auxiliary subdomain, such as KRAB-B [7–9], KRAB-BL [1]**,** KRAB-b [10] or KRAB-C [11]. The canonical model of function proposes that KRAB-ZNF proteins mediate transcriptional repression by recruitment of the tripartite motif protein TRIM28 (synonyms KAP1, TIF1β, KRIP-1, SMP1) and its associated proteins and activities to genomic loci specified by the ZNF DNA-binding motifs [4, 12, 13]. The KRAB domain was proposed to interact with sequences from within the N-terminal RING finger/B-box/coiled-coil domain region of TRIM28 [14–16]. Novel structural studies pinpointed the major interaction to the central coiled-coil domain of a TRIM28 dimer [17, 18]. TRIM28, in turn, serves via its C-terminal half as a hub for chromatin-modifying proteins that include the histone methyltransferase SETDB1, the nucleosome remodeling and deacetylase complex (NuRD) [19, 20] and heterochromatin HP1 proteins [21]. Automodification of TRIM28 with small ubiquitin-like modifier peptides (SUMO) stabilizes the interactions to SETDB1 and the NuRD component CHD3, SETDB1 trimethylates histone H3 lysine 9 (H3K9me3) and members of the HP1 protein family further bridge the complex with chromatin by simultaneously binding to TRIM28 and the modified H3K9me3 histone tail [22–24]. Ultimately, these processes establish heterochromatin-like silenced chromatin regions.

As a consequence of the canonical model for KRAB-ZNF transcription factors, KRAB domain function in respect to transcriptional regulation is usually investigated in heterologous reporter assays using fusions of the domain under study to known DNA-binding motifs [8, 12] and by characterizing its interaction with TRIM28. Numerous KRAB-ZNF proteins have been shown to conform to the canonical model of transcriptional repression through TRIM28 recruitment. However, several studies report the involvement of KRAB-ZNF proteins in transcriptional activation [25–28] and some members failed to stably interact with TRIM28 despite trans-repressor activity [29, 30]. Analyses of conserved residues and mutants of KRAB-A identified amino acid motifs important for canonical KRAB-A function [8, 30, 31]. The roles of the auxiliary subdomains are unclear. However, the KRAB-B subdomain itself does not display repressor activity, but has been shown to enhance the KRAB-A function through an unknown mechanism [9].

Usually, the KRAB subdomains are encoded by individual exons while the whole array of tandem zinc finger motifs is encoded by a single exon [7]. Subsets of the (KRAB)-ZNF proteins contain additional conserved sequences at their amino termini, such as the SCAN domain and the “domain of unknown function 3669” (DUF3669) [4, 32]. The SCAN domain has been described as protein oligomerization domain [33] and SCAN-KRAB-ZNF proteins have been postulated to confer transcriptional repression independent of TRIM28 [29]. The DUF3669 protein family has initially been defined in the PFAM database (http://pfam.xfam.org/family/PF12417) and is characterized by a consensus motif of a rather low overall conservation. Worth noting, the database lists members from fungi to all metazoans.

The human genome contains seven genes encoding DUF3669-containing proteins that are clustered at chromosome locus 7q36.1. Six of them, ZNF212, ZNF282, ZNF398, ZNF746, ZNF777 and ZNF783, encode KRAB-ZNF proteins [32]. Although named ZNF767, the seventh gene potentially gives only rise to a protein just consisting of DUF3669 without any other known domain. In public databases, ZNF767 is considered to be a pseudogene. The KRAB-A subdomains of the DUF3669-containing ZNF proteins do not conform to conserved sequence motifs establishing canonical KRAB-A. In addition, ZNF282, ZNF777 and ZNF783 are evolutionary older than most other KRAB-ZNF genes in that they go back to sauropsids [32, 34].

ZNF746 (synonym PARIS) has been implicated in neurodegenerative disease. It was reported that ZNF746 can silence the promoter of the peroxisome proliferator-activated receptor gamma coactivator-1a (PGC-1α) [35] and of the transketolase (TKT) gene [36]. PGC-1α is a master regulator for mitochondrial biogenesis while TKT constitutes a key enzyme in the pentose phosphate pathway. The ZNF746 protein level itself is controlled by parkin whose E3 ubiquitin ligase activity marks ZNF746 for proteasomal degradation in a phosphorylation-dependent manner [37]. In Parkinson’s disease, parkin loss-of-function mutants lead to ZNF746 overexpression with disturbance of mitochondrial homeostasis and energy metabolism and concomitant loss of dopaminergic neurons [35, 38, 39]. The sumoylation status of ZNF746 modulates its transcriptional repressor activity towards the PGC-1α promoter in a complex, not fully understood manner [40]. Interactome analysis of ZNF746 argues for a role in the regulation of rRNA expression [41]. ZNF746 has also been implicated in colon cancer [42], lung cancer [43] and bladder cancer [44].

Prior to the formal definition of DUF3669 in the PFAM database, ZNF282/HUB1 and ZNF398/ZER6 were described as ZNF proteins whose unusual KRAB domains can transactivate their target genes as an isolated domain [45, 46]. Notably, protein regions that we can now assign to DUF3669 have been shown to have a repressive effect on gene expression (ZNF282/HUB1, [46]) or to inhibit protein-protein interaction with estrogen receptor alpha (ZNF398/ZER6, [45]). Individual domains of ZNF777 have not been characterized, but a negative effect on cell proliferation was found to be independent of the presence of the KRAB domain [47].

Here, we studied the role of the N-terminal DUF3669 and KRAB domains of two ZNF746 isoforms and ZNF777 for transcriptional repression. We found dependencies of repressor function on the KRAB domain configuration (truncated, full-length, sequence-optimized by mutation), the DUF3669 protein segment and adjacent sequences containing a SUMO acceptor lysine. The magnitude of the contribution of a KRAB domain to repression paralleled the extent of its interaction with TRIM28. Our data establish the DUF3669 sequences of ZNF746 and ZNF777 as novel protein-protein interaction modules that confer oligomerization.

## Results

In this study, we focused on a subgroup of KRAB-ZNF transcription factors that in addition to KRAB domain and zinc finger DNA binding sequences contain a further conserved protein region, the “domain of unknown function 3669” (DUF3669). Our study of ZNF746 and ZNF777 investigated the contribution of cognate KRAB and DUF3669 domains to transcriptional modulation including the formation of protein complexes. ZNF746 attracted our interest because of its involvement in Parkinson’s disease [35] and ZNF777 because of its old evolutionary age [32, 34].

### DUF3669 sequences are evolutionary highly conserved in amniotes

**Fig. 1:**
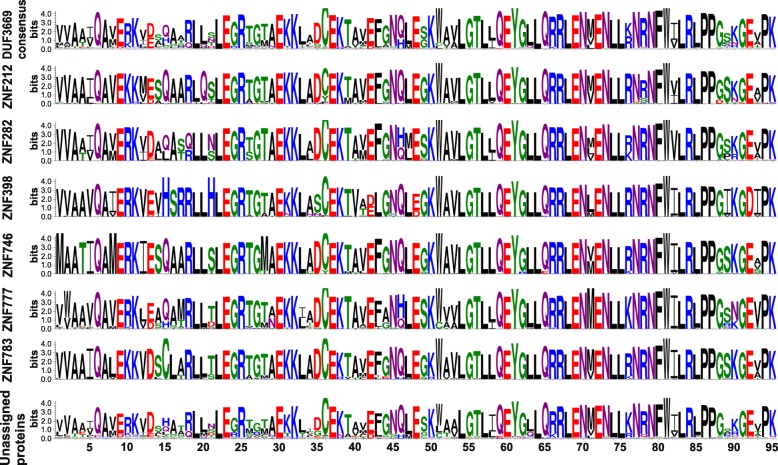
Compilation of amino acid sequence logos for DUF3669 domain-encoding genes from mammals, birds and reptiles. The x-axis indicates the position within the domain and the y-axis denotes the information content (bits) for each amino acid. The height represents the frequency of each amino acid at a certain position within the tested sequence set. The overall DUF3669 consensus sequence at the top was derived from all DUF3669-containing proteins identified in amniotes (*n* = 538. The other DUF3669 domain logos represent the ortholog groups of six of the seven human DUF3669-encoding KRAB-ZNFs (ZNF212 *n* = 49; ZNF282 *n* = 99; ZNF398 *n* = 31; ZNF746 *n* = 59; ZNF777 *n* = 90; ZNF783 *n* = 33) and proteins that could not unequivocally attributed to any human ortholog group (“unassigned proteins”; *n* = 176). ZNF767 was only found as a single human pseudogene and is not depicted here. See Additional file 1 for a complete compilation

The original definition of DUF3669 is based on sequence alignments and model building in the PFAM database (protein domain identifier PF12417). Probably due to the inclusion of low homology sequences from fungi and all metazoan taxa, the resulting domain model looks rather degenerated with a low number of strongly conserved amino acid residues just at the borders. We decided to reach for a new, more concise domain model that was based on the DUF3669 sequences of the seven human members. We compiled all orthologs using public database resources and reciprocal BLAST searches and built an overall consensus DUF3669 domain logo as well as individual ortholog group logos (Fig. 1). Orthologs of the human DUF3669-containing proteins were only found in amniotes from reptiles and birds to mammals, but not in amphibians. Thus, sauropsids are the evolutionary oldest taxa encoding DUF3669 sequences according to our definition. Our model describes an extremely conserved consensus polypeptide of 95 amino acids. A comparison between the sequence logos of different ortholog groups, named after the human protein, showed a striking similarity among them. ZNF282 and ZNF777 orthologs clearly exist outside mammals in reptiles and birds, while only one protein in a turtle species could be confidently assigned to ZNF212 (see compilation in Additional file 1). We were not able to find unambiguous examples for ZNF398, ZNF746 and ZNF783 orthologs outside mammalian species. This suggests that the latter three members emerged more recently than the other DUF3669-containing KRAB-ZNFs during evolution. ZNF767 appears to be a human-only pseudogene (e.g. NCBI Gene ID: 79970) although its short sequence of almost only DUF3669 residues may possibly prevent clear-cut ortholog assignments. Thus, DUF3669 is an evolutionary highly conserved domain existing since the emergence of sauropsids about 310 million years ago. The high sequence conservation during evolution points to essential protein functions inherent in DUF3669 that appear to be shared between different ortholog groups with this domain.

### Contribution of the aminoterminal domains of DUF3669-KRAB-ZNF proteins to transcriptional repression

The full-length ZNF746/PARIS was reported to repress transcription about 2fold in a heterologous reporter assay [35]. However, the domains contributing to this activity had not been elucidated till now. In addition, we noticed that previous studies focused on an ZNF746 isoform that contains an N-terminally truncated KRAB-A subdomain. Interestingly, gene databases list sequence information on another isoform that displays a full-length KRAB-A. Thus, we distinguished in our experiments constructs of isoform ZNF746a (15 amino acid truncation; UniProtKB: Q6NUN9) and isoform ZNF746b (UniProtKB: A0A2R8YDQ5). The cloning of ZNF746b-derived constructs by RT-PCR using human testis RNA as input provided evidence for the existence of the full-length KRAB-A isoform.

**Fig. 2:**
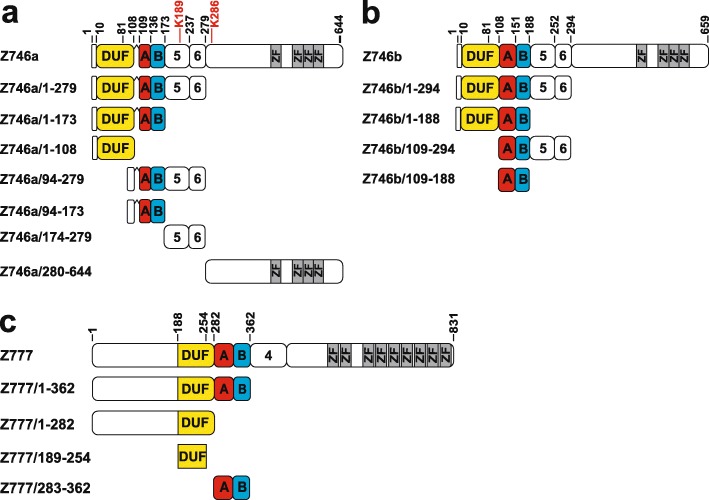
Representations of the primary protein structures encoded by the various constructs employed in this study. Shown are the full-length proteins and the truncated versions along with the names of the constructs/protein pieces used throughout the manuscript (drawn to scale). These regions were expressed as fusion proteins with Gal4 or GST, respectively, depending on experimental goals. Each box with rounded corners denotes the protein region encoded by a separate exon. The numbers above the cartoon of the longest form correspond to the amino-acids positions in the respective full-length protein. Middle exons without known conserved domain are designated by their number. The known protein domains are labeled, i.e. DUF3669 (DUF, yellow), KRAB-A (“A”, red), KRAB-B (“B”, blue), C2H2 zinc finger motif (ZF, grey). **a** Canonical ZNF746 isoform designated Z746a (represented by UniProtKB Q6NUN9) containing a truncated KRAB-A domain indicated by a gap in the depiction for highlighting this fact. **b** ZNF746 isoform designated Z746b (represented by UniProtKB A0A2R8YDQ5) with full-length KRAB-A domain. (**c**) ZNF777 protein (UniProtKB Q9ULD5–2)

**Fig. 3:**
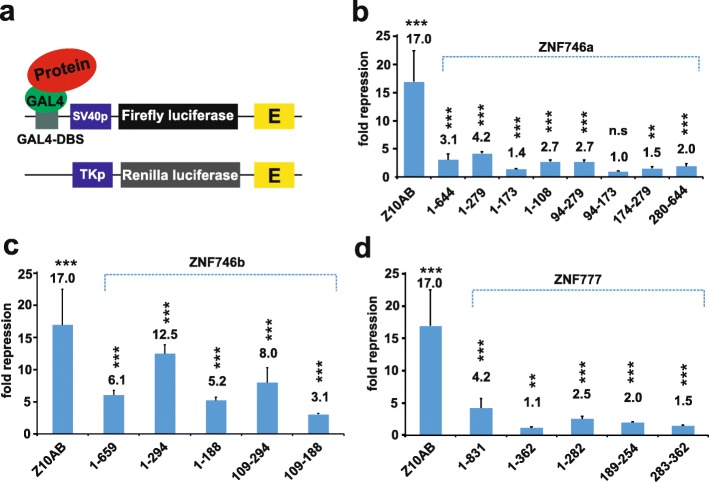
Contribution of different protein domains to the transcriptional repressor activities of ZNF746 and ZNF777. Heterologous reporter assay data from human HeLa cells using effector plasmids that encode fusions of the indicated protein segments with the Gal4 DNA-binding domain. The fusion of the ZNF10 KRAB domain (Z10AB) was used as a positive control and reference for potent repressor activity. This reference is included in each subfigure plot. **a** Illustration of assay; Firefly luciferase reporter gene cassette with Gal4 DNA binding sites (Gal4-DBS) upstream of a strong viral SV40 promoter and 3′ enhancer elements (“E”); *Renilla* luciferase cassette with strong viral HSV TK promoter, which does not have GAL4-DBS for normalization purposes. **b** Repressor potency of isoform ZNF746a and its selected truncations. **c** Repression factors of isoform ZNF746b and its selected segments. **d** Repressor activity of ZNF777 and its selected truncations. Bar plots represent normalized mean repression factor values ± STDEV of at least six biological replicates from three independent experiments relative to experiment-specific values for the Gal4-DBD alone. Asterisks indicate results of a two-tailed unpaired t-test (* *p* < 0.05, ** *p* < 0.01, *** *p* < 0.001)

First, we assessed the transcriptional repression capacities of the different protein regions (Fig. 2 a, b) of ZNF746 in classical heterologous reporter assays in HeLa cells (Fig. 3a). Our initial results confirmed that the full-length ZNF747a isoform was able to repress reporter gene expression. The repression factor reached 3.1fold, a value similar to the above-cited work, but rather weak compared to a bona fide KRAB-AB domain like ZNF10-AB [8] that we used as a positive control (Fig. 3b). Nevertheless, we consider this repressive activity significant since it was robust and well above the values of two negative controls, protein segments of PRDM9, namely the KRAB-like domain of PRDM9 (P9/24–97) and the extended N-terminal KRAB-SSXRD-PRSET segment of PRDM9 (P9/24–363) with *p* values < 0.01 in a two-tailed unpaired t test. Those were shown to be inactive in repression in HeLa cells reaching values between 0.7 and 1.3 (see Additional file 2 and reference [48]). Further, the PRDM9 KRAB domain was reported to not act like a canonical KRAB domain since it did not interact with TRIM28 ([49, 50]). When we analyzed individual N-terminal ZNF746 protein segments, repression reached even 4.2fold for the segment that included DUF3669, truncated KRAB and adjacent residues encoded by exons 5 and 6 (construct ZNF746a/1–279). However, the KRAB domain itself appeared not to contribute to this activity since ZNF746a/94–173 did not have any impact on reporter gene activity. We chose to include amino acids 94–108 in this construct to bring the truncated KRAB-A sequence to a similar size as a canonical KRAB-A in case of steric effects and to avoid overlooking a possible impact of these neighboring residues even if they are not conserved among KRAB domains. In contrast, the ZNF746a/1–108 protein encoding the DUF3669 alone accounted for most of the repressive potential (factor 2.7) and exon5–6 encoded residues (ZNF746a/174–279) were necessary to bring this up to the 4.2fold of the whole N-terminal configuration. However, the repression activities of segments made of individual domains did usually not simply add up when combined. For example, ZNF746a/94–173 (KRAB, value 1, i.e. no repression) and ZNF746a/174–279; value 1.5, i.e. no significant repression activity) act together in a synergistic manner to result in a low, but robust 2.7 fold repression effect. Such data point to the importance of the whole N-terminal domain configuration for the repression function. The C-terminal half of ZNF746 including the zinc finger motifs had only a very weak repression activity of 2fold. Altogether, the results indicated that DUF3669 alone can confer weak, but reproducible, transcriptional repression and the full repression potential of the N-terminal half of ZNF746a resided in the DUF3669 plus amino acids encoded by exon5/6 of the protein.

We next turned our analysis to the N-terminal configuration that contains the full-length KRAB-A domain represented in isoform ZNF746b. Our results emphasized a general gain in repression potential compared to isoform ZNF746a for each of the analogous constructs, from the full-length ZNF746b to the KRAB domain (ZNF746b/1–188) alone (two-tailed unpaired t-test *p*-values < 0.001 for each comparison). The maximal value was reached with the whole N-terminal configuration of DUF3669/KRAB-AB/exon5–6 (ZNF746b/1–294) reaching a repression factor of 12.5 (Fig. 3c). This increase compared to the ZNF746a segments can be attributed to the full-length KRAB-AB since this domain alone exerted moderate, but robust 3.1fold repression activity. Yet, this activity is still much less strong than the factor of 17fold displayed by ZNF10 KRAB-AB. Overall, the data revealed that the full-length KRAB-A subdomain of isoform ZNF746b boosts the overall repressive potential of ZNF746.

Next, we evaluated whether ZNF777, a paralog of ZNF746 with an N-terminal domain configuration of DUF3669 and full KRAB (Fig. 2c), displayed similar properties with respect to transcriptional repression. We focused on the sequences encoding the DUF3669 and KRAB domains of ZNF777. Full-length ZNF777 was, like ZNF746a, a weak transcriptional repressor in our assay system (4.2fold, ZNF777/1–831; Fig. 3d). The testing of the individual ZNF777 segments corroborated the initial finding for ZNF746a. Again, DUF3669 contributed more to the overall repression potential than the KRAB-AB domain. The whole exon-derived sequence including the DUF3669 (ZNF777/1–282) and a narrowed down DUF3669 segment (ZNF777/189–254; this part would fit the PFAM consensus definition of DUF3669) showed 2.5 and 2fold downregulation of reporter gene activity, while the KRAB-AB (ZNF777/283–362) only downregulated reporter gene expression by 1.5fold. Possibly, further repression potential, sufficient to close the gap to the activity of full-length protein, might reside, like in ZNF746, in the C-terminally adjacent sequences of the ZNF777 KRAB domain. Although complete in length, the ZNF777 KRAB-AB domain behaved more like the truncated KRAB of isoform ZNF746a than the full KRAB of isoform ZNF746b. Differences at individual residues in their KRAB-A domain as well as in their cognate KRAB-B domains that might boost KRAB-A-repressive activity to a variable extent [9] possibly explain this observation.

Altogether, our results show that the N-terminal configuration of DUF3669/KRAB-AB/exon5–6-residues determines the repressive capacity of ZNF746. A full KRAB-AB domain in isoform ZNF746b enhances the repression activity, but nevertheless requires DUF3669 and exon5–6 residues for its full potential. The contribution of DUF3669 to transcriptional repression was significant and also observed for ZNF777.

### Dependency of repressor activities of DUF3669-KRAB-ZNF proteins on TRIM28 and SETDB1

**Fig. 4:**
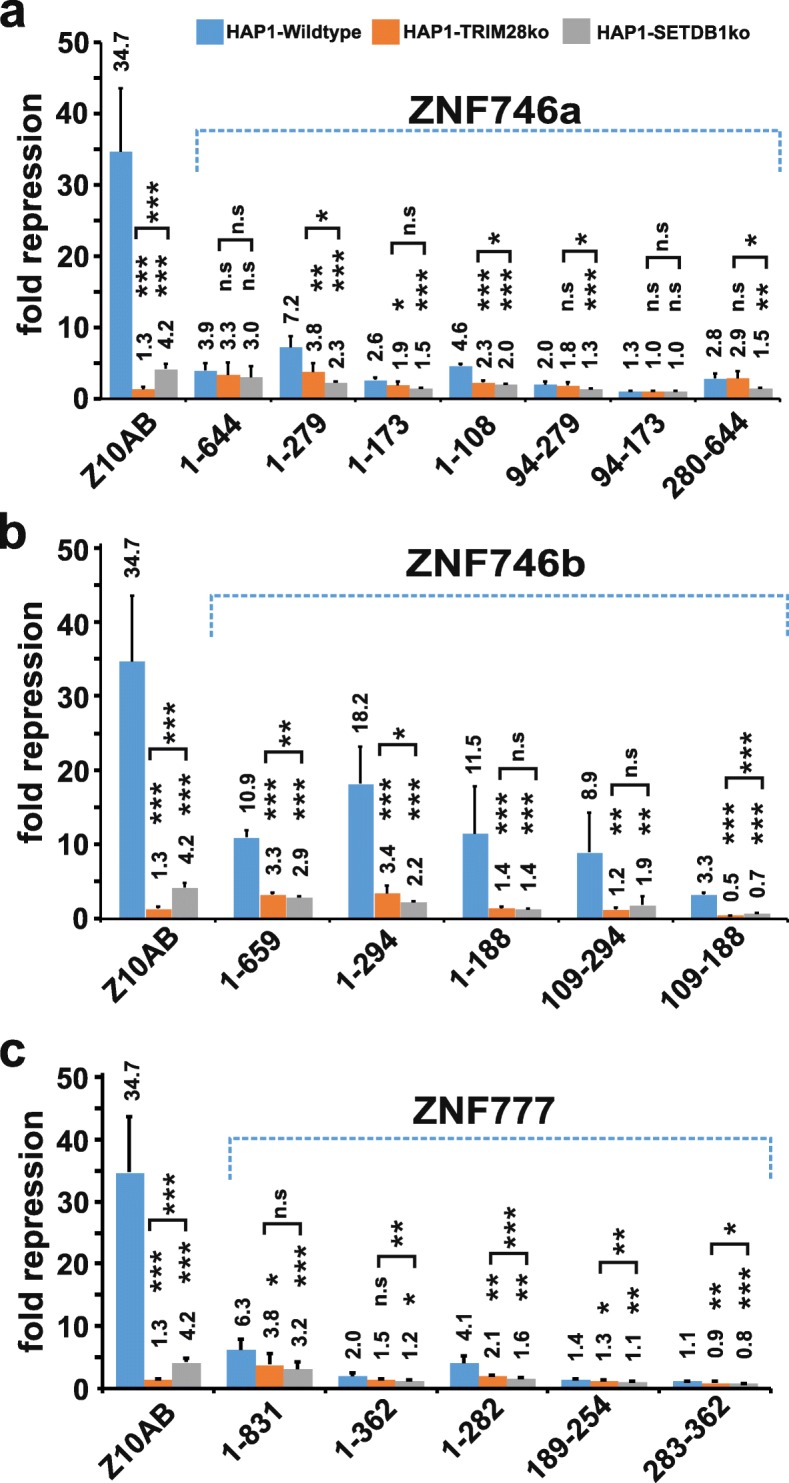
Dependency of the transcriptional repressor functions of ZNF746 and ZNF777 on TRIM28 and SETDB1. Results of dual luciferase reporter assays using Gal4 DNA-binding domain fusions of the indicated protein segments. The ZNF10 KRAB domain (Z10AB) served as positive control for dependency on TRIM28 and SETDB1. **a** ZNF746a-derived protein fragments. **b** ZNF746b-derived protein fragments. **c** ZNF777-derived protein fragments. Bar plots depict normalized mean repression factor values ± STDEV of at least six biological replicates from three independent experiments relative to experiment-specific and cell-type specific values for the Gal4 DNA-binding domain alone. Results for wildtype HAP1 cells (HAP1-WT; blue bars), TRIM28-knockout HAP1 cells (TRIM28ko, orange bars) and SETDB1 knockout HAP1 cells (SETDB1ko, grey bars). Asterisks indicate results of a two-tailed unpaired t-test (* *p* < 0.05, ** *p* < 0.01, *** *p* < 0.001; n.s. = no statistical significance, i.e. *p* > 0.05) when comparing knockout with wildtype data of a protein fragment

TRIM28 and SETDB1 proteins are mechanistically required to achieve transcriptional repression initiated by recruitment of canonical KRAB domains to DNA (see introduction). We therefore studied TRIM28 and SETDB1 dependencies of the repressor function of the ZNF746 isoforms in wildtype versus TRIM28 knockout (KO) and SETDB1 KO HAP1 cell models (Fig. 4). The almost complete loss after TRIM28 KO and strong drop after SETDB1 KO of the repression potential of the ZNF10 KRAB domain illustrates the potential and validity of this approach. Based on the repression values of the two negative controls, fusions of Gal4 to the KRAB domain of PRDM9 (P9/24–97) and the extended N-terminal KRAB-SSXRD-PRSET segment of PRDM9 (P9/24–363) (see Additional file 2), we consider repression factors above 2 to represent valid repressing events. While the absolute repression factors reached higher values in HAP1 wildtype versus HeLa cells, reporter assays in both cell types gave approximately equivalent results. In particular, the segments with the whole N-terminal configuration of DUF3669/KRAB/exon5–6 of both ZNF746 isoforms displayed once again the highest degree of downregulation (Fig. 4a: ZNF746a/1–279 by factor 7.2; Fig. 4b: ZNF746b/1–294 by factor 18.2). In cells with TRIM28 ablation, repression activities of ZNF746a/1–279 decreased by about 50% to 3.8fold while those of ZNF746b/1–294 with its complete KRAB domain went down by even 80% to a similar remaining factor of 3.4fold. The contribution of TRIM28 to the repressive effect turned out to be higher for the more canonical complete KRAB domain. Yet, the residual activity, in particular when compared to the almost complete loss of repression potential of ZNF10-KRAB, clearly indicated the participation of TRIM28-independent components providing transcriptional impact. The effect of SETDB1 ablation was moderately stronger and residual repression activity lower, but not completely lost (2.3fold for ZNF746a/1–279; 2.2fold for ZNF746b/1–294). Interestingly, residual activities were on a lower level compared to the ZNF10-KRAB domain (4.2fold). These results open up the possibility that SETDB1 additionally contributed by other mechanisms than by the usual indirect recruitment through TRIM28 in the canonical KRAB/TRIM28/SETDB1 model. The other N-terminal segments were altogether in line with these results as described above, just at lower levels of repression activity. Reduction of the repressive potential of ZNF746 segments lacking KRAB sequences in the DUF3669 construct (ZNF746/1–108) and the C-terminal half (ZNF746/280–644) in the absence of TRIM28 or SETDB1 might be interpreted as non-canonical, KRAB-independent mechanisms of TRIM28 and SETDB1. When we analyzed the full-length ZNF746a protein (Fig. 4a; ZNF746a/1–644) the repressive activity of 3.9 fold was only slightly decreased to 3.3fold (TRIM28 KO) and 3fold (SETDB1 KO) and statistical tests did not reach *p*-values below 0.05. In contrast, the increases in repression potential of full-length ZNF746b and all its derivatives with complete KRAB-A box were strongly dependent on TRIM28 and SETDB1 because they disappeared in the respective KO cell lines (p-values wildtype vs TRIM28ko or SETDB1ko cells always at least < 0.01).

Analogously, we analyzed our N-terminal fragments containing DUF3669 and KRAB sequences of ZNF777 in HAP1 cell models of depleted TRIM28 and SETDB1. We observed overall the same tendencies as for ZNF746, decreased repressive forces after ablation of TRIM28 and SETDB1, with slightly higher impact of SETDB1 (Fig. 4c). This was again true not only for segments including the KRAB domain, but also for only DUF3669-containing fragments. The strongest effect was recorded for the polypeptide encoded by the whole exon 1 (ZNF777/1–282) which decreased in repression activity from 4.1 in HAP1 wildtype to 2.1 in TRIM28 KO and 1.6 in SETDB1 KO cells. Full-length ZNF777 (ZNF777/1–831) showed similar reduction in its 6.3fold repression potential by about 40% (TRIM28 KO) and 50% (SETDB1 KO).

In conclusion, our DUF3669-KRAB-ZNF proteins contain repressive activities that can be attributed in part to TRIM28- and SETDB1-dependent mechanisms, not necessarily, however, following a canonical manner involving a classical KRAB/TRIM28/SETDB1-complex. Nevertheless, impact of TRIM28 or SETDB1 loss on repression potential was the strongest for the fragments that included the KRAB domain with the highest repressor activity, namely that of the ZNF746b isoform.

### Evaluation of the association of aminoterminal portions of DUF3669-KRAB-ZNF proteins and TRIM28 in shared complexes

**Fig. 5:**
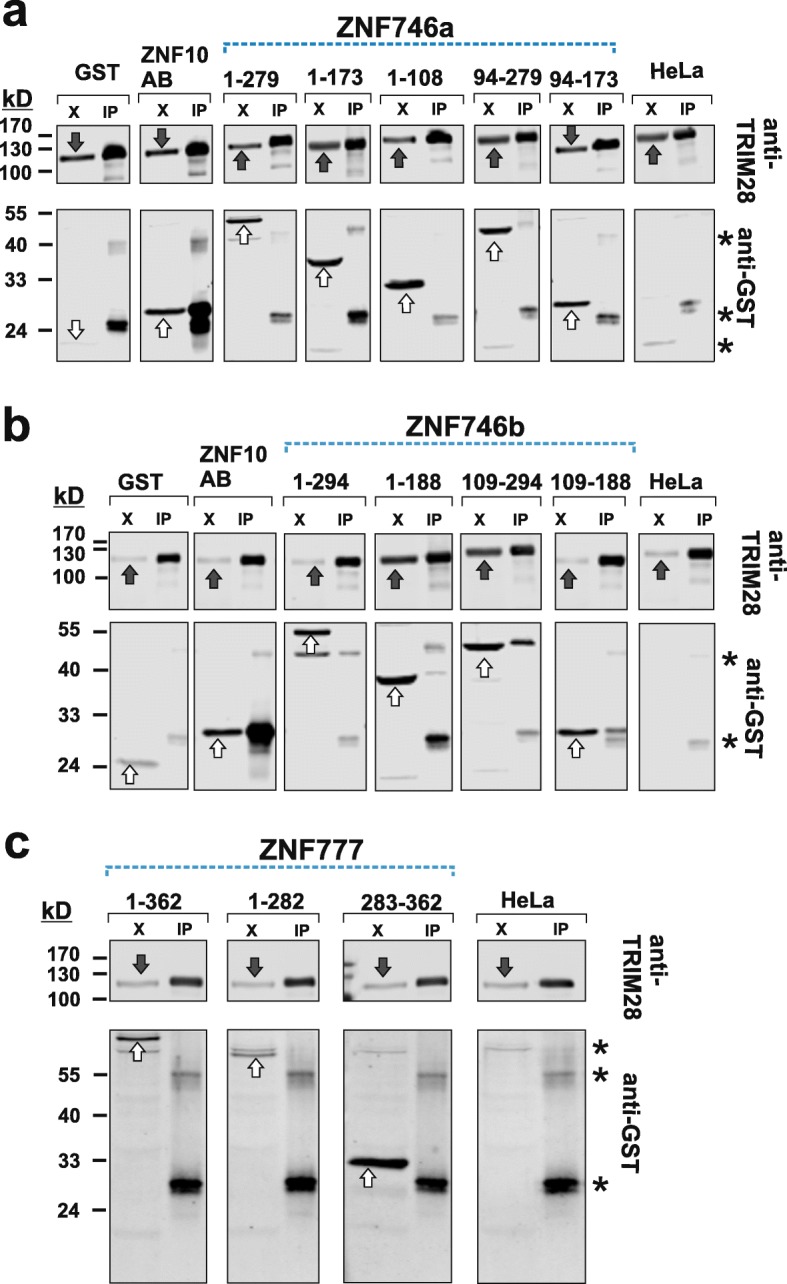
Analysis of the interaction between ZNF746 or ZNF777 protein fragments and endogenous TRIM28. Extracts from HeLa cells after transient expression of GST/GST-ZNF fusion constructs were subjected to immunoprecipitation using TRIM28 as bait. Input protein extracts (“X”) and eluted immunoprecipitates (“IP”) were analyzed by Western blotting. The blots were probed with monoclonal antibodies against TRIM28 and rabbit polyclonal antibodies against GST, followed by respective secondary antibodies with different fluorescent tags. Images represent black/white representations of cropped regions (see Methods) informative to evaluate TRIM28 precipitation (upper part) and GST/GST-fusion protein co-immunoprecipitations (lower parts) from the same lanes. White block arrows indicate GST/GST-fusion protein signals and the black block arrows point to TRIM28 bands in the input cell lysate lanes. GST alone was used as a negative control whereas the KRAB domain of ZNF10 served as positive control. Further controls were obtained by mock immunoprecipitation from non-transfected HeLa lysates (label “HeLa”). **a** Results for Z746a-derived GST fusions. **b** data of Z746b-derived GST fusions. **c** analysis of Z777-derived GST fusions. Asterisks indicate unspecific bands

To investigate whether the partial functional dependencies on TRIM28 described above coincided with the cellular interaction of ZNF746 and ZNF777 with TRIM28 in protein complexes we performed co-immunoprecipitation assays in HeLa cells. As positive control for efficient and stable complex formation with TRIM28 we used once more the KRAB domain of ZNF10. Endogenous TRIM28 in HeLa cells was used as bait to analyze the enrichment of fusion proteins between the protein fragments under study and GST as tag (Fig. 5). The analysis in HeLa cells showed that none of the investigated N-terminal segments of the ZNF746a, the isoform with truncated KRAB-A, was able to stably associate with TRIM28 (Fig. 5a). In contrast, protein fragments containing the full-length KRAB-A domain of isoform ZNF746b within the N-terminal segments, either containing KRAB-AB alone (ZNF746b/109–188), DUF3669/KRAB (ZNF746b/1–188) or KRAB/exon5–6-encoded residues (ZNF746b/109–294), were complexed to TRIM28 (Fig. 5b). However, the extent of enrichment was clearly much lower than for the ZNF10 KRAB domain arguing that the stability of the complex and/or affinity of the binding partners were inferior for ZNF746b. Surprisingly, the whole N-terminal segment made of DUF3669/KRAB/exon5–6 that showed the highest repression potential (see above) was not at all co-immunoprecipitated with TRIM28. This result was reproducible and was corroborated when we used anti-GST antibodies for immunoprecipitation of this ZNF746b segment which failed to enrich endogenous TRIM28 (data not shown). We speculate that the tertiary structure or folding issues of the GST fusion with this ZNF746b/1–294 fragment might have caused this failure. Finally, the ZNF777 N-terminal segments did not show any potential for stable association with endogenous TRIM28 (Fig. 5c).

In summary, our results emphasize a gain of possible new mechanisms of action for the ZNF746b compared to the ZNF746a isoform or vice versa a loss of functionality for ZNF746b. Obviously, the presence of a complete KRAB-A domain in ZNF746b enables a more stable interaction with TRIM28. However, this association is still inferior compared to the complex of a bona fide canonical KRAB domain like the ZNF10-AB domain, a result that fit the data on transcriptional repression activities.

### Functional improvement of ZNF746b KRAB-AB by mutation to a conserved acidic residue

**Fig. 6:**
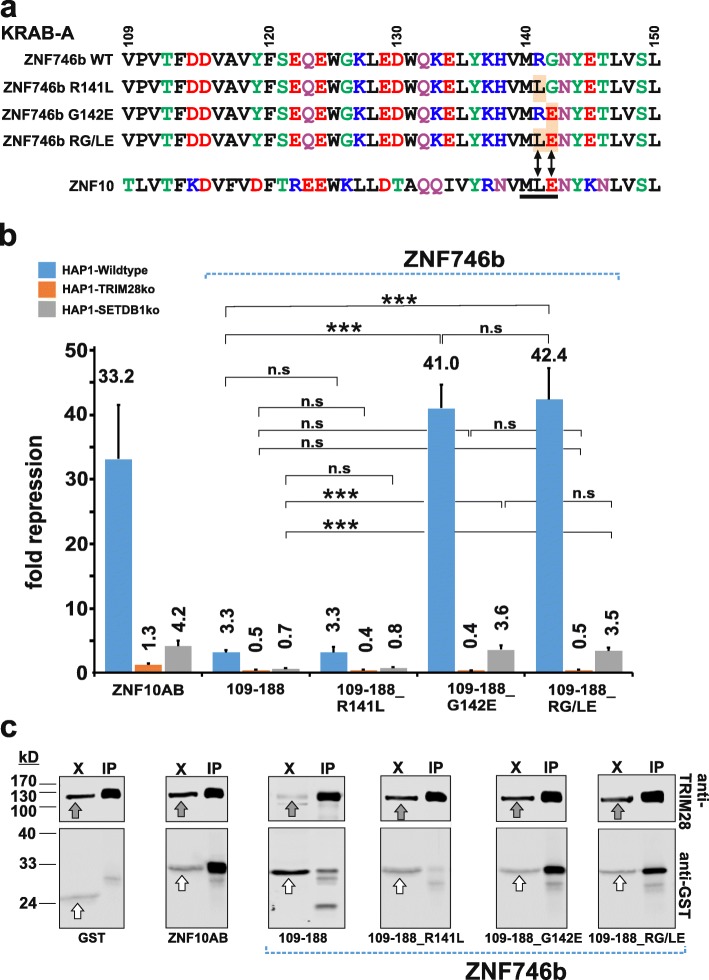
Mutation to a conserved glutamate residue in ZNF746b KRAB-A potentiates its repression activity and TRIM28 interaction. **a** Multiple alignment of the KRAB-A amino acid residues of ZNF746b and three ZNF746b-KRAB-A mutants, R141L, G142E and RG/LE (positions in the complete protein indicated on top and mutated residues highlighted) along with the canonical sequence of ZNF10. The conserved canonical “MLE” motif is underscored in the ZNF10 sequence. **b** Comparison of transcriptional repression activities of wild type KRAB-AB of ZNF746b, and the three configurations with the indicated mutated residues in KRAB-A in HAP1 wild type (blue bars), SETDB1ko (orange bars), and TRIM28ko cell lines (grey bars). The KRAB domain of ZNF10 (Z10AB) is used as positive control. Results of dual luciferase assays (see Fig. 3) presenting mean repression factor values ± STDEV of at least six biological samples from three independent experiments. Asterisks indicate results of a two-tailed unpaired t-test (* *p* < 0.05; ** *p* < 0.01; *** *p* < 0.001; n.s. = not significant, i.e. *p* > 0.05) (**c**) Analysis of complex formation of GST-KRAB-AB fusion proteins with TRIM28 in HeLa cells using a co-immunoprecipitation/Western blot assay with endogenous TRIM28 as bait as described in Fig. 4. Grey block arrows point to endogenous TRIM28 signals in the lysate lanes (X), and white block arrows point to the GST/GST fusion bands in the lysate lanes (X)

Inspection of an alignment of KRAB-A amino acid residues of ZNF746b and ZNF10 as a representative of the group with known canonical KRAB-A subdomain highlighted a difference in the conserved “MLE” motif known to be important for KRAB repressor activity [8, 30]. Namely, positions at ZNF746b/140–142 read “MRG”, i.e. there are clear charge differences of the amino acid side chains with a positively charged arginine instead of the nonpolar leucine and a small glycine instead of the acidic glutamic acid (Fig. 6a). This comparative analysis motivated us to investigate whether the amino acids R141 and G142 in ZNF746b KRAB-A might be responsible for the considerably weaker repression potential and lower association power to TRIM28 compared to ZNF10 KRAB-A. We therefore generated mutants that replaced either one residue or both together and performed heterologous reporter assays to measure transcriptional potency and co-immunoprecipitation experiments to look at TRIM28 association.

When tested in HAP1 wildtype, TRIM28 KO and SETDB1 KO cells, the R141L mutation behaved like the wildtype ZNF746b KRAB-AB domain (ZNF746b/109–188; Fig. 6b). Most importantly, this mutant did not improve at all the overall repression activity in wildtype cells. In contrast, a huge gain in repressor potency to levels surmounting even the ZNF10 KRAB-AB positive control was documented when the glycine residue was mutated to glutamic acid and tested in HAP1 wildtype cells (construct ZNF746b/109-188G142E). This increase was completely dependent on the presence of TRIM28. Loss of SETDB1 was also strongly detrimental to the gain of repressor activity. Yet, unlike after TRIM28 depletion, we still measured residual repressor activity in SETDB1-depleted HAP1 cell models (Fig. 6b). This situation was reminiscent of the behavior of the ZNF10 KRAB domain and points to transcriptional mechanisms contingent on TRIM28, but independent of SETDB1. The double mutant of ZNF746b-KRAB, RG141,142LE displayed the same properties as the single G142E mutant without any further increase in repressor potency or any difference in control by TRIM28 and SETDB1. In summary, the data indicate that already one amino acid change from glycine (G) to glutamic acid (E) in a KRAB-ZNF transcription factor, achievable by the exchange of one single nucleotide within the codons, can have dramatic consequences for transcriptional gene regulation.

We next confirmed our functional data by determining the association of wildtype and mutant ZNF746b-KRAB-AB domains with cellular TRIM28 in HeLa cells in co-immunoprecipitation assays. The R141L mutant of ZNF746b-KRAB did not improve association with TRIM28 compared to the wildtype construct (ZNF746b/109–188; Fig. 6c). The faint band on the blot even suggested lower enrichment for this mutant. In contrast, the G142E mutant was efficiently pulled down using the endogenous TRIM28 as bait, to an extent considerably higher than the wildtype ZNF746b-KRAB and similar to the ZNF10 bona fide KRAB domain. The double mutant RG141,142LE of ZNF746b-KRAB did not lead to further enhancement of association with TRIM28. Overall, our experiments validate the impact of the replaced glutamic acid residue of the conserved “MLE” motif in canonical KRAB-A sequences as the reason for the considerably weaker transcriptional repression potency and for the reduced association of the ZNF746b KRAB domain with TRIM28. The higher repressor activity was due to stronger binding to TRIM28.

### Lack of effect of DUF3669 on the repressor potency of a canonical KRAB domain

**Fig. 7:**
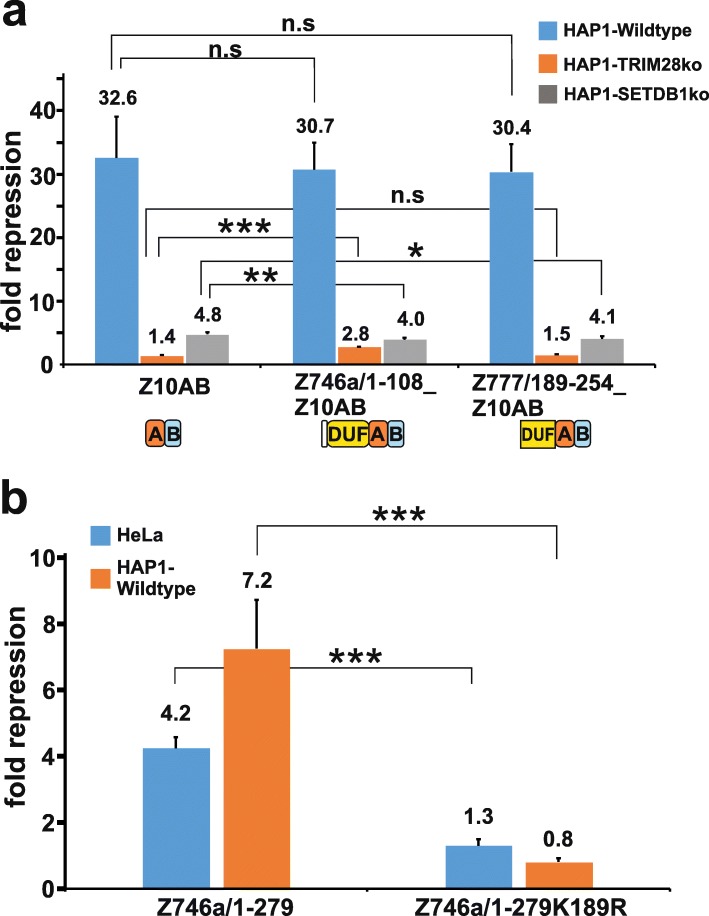
Analysis of transferability of DUF3669 and the potential impact of sumoylation. **a** Negligible impact of DUF3669 domains from ZNF746a and ZNF777 on the repressor activity of ZNF10 KRAB-AB. Wildtype ZNF10-AB and fusions of ZNF10-AB to DUF3669 domain segments from ZNF746a or ZNF777 were analyzed by dual luciferase assays in HAP1 wildtype, SETDB1 and TRIM28 knockout cells as in Fig. 4. **b** Mutation of a major SUMO-acceptor lysine (K189R) in the Z746a/1–279 fragment abolished its repressor activity in HeLa cells (blue bars) and HAP1 wildtype cells (orange bars). Bars visualize mean repression factor values ± STDEV of at least six biological samples from three independent experiments. Asterisks indicate results of a two-tailed unpaired t-test (*** *p* < 0.001; n.s. = not significant, i.e. *p* > 0.05) for the comparison of the DUF3669 fusions with the KRAB domain of ZNF10 (Z10AB)

To investigate if DUF3669 can inherently support the transcriptional repressive potential of a canonical KRAB domain, DUF3669-containing fragments of ZNF746 (ZNF746a/1–108) and ZNF777 (ZNF777/189–254) were expressed as chimeras with the ZNF10 KRAB-AB at the C-terminus. Experiments in HAP1 wildtype, TRIM28 KO and SETDB1 KO cells indicated that the properties of ZNF10-KRAB-AB did altogether not change (Fig. 7a). Neither the absolute repression potential in wildtype cells nor the dependency on TRIM28 or SETDB1 was visibly modified. After depletion of TRIM28 there was more residual repressor activity for the ZNF746-DUF3669 fusion than for ZNF10-AB alone. However, this effect was weak and not found with the ZNF777-DUF3669 fusion. Overall, our results argue that DUF3669 domains act in a context-dependent manner and do not contain intrinsic abilities to modulate the repressor function of canonical KRAB domains.

### A potential SUMO acceptor site is critical for the transcriptional repression activity of the aminoterminal ZNF746a protein segment

ZNF746 contains two primary sumoylation sites (lysines K189 and K286 in isoform ZNF746a). Their mutation leads to a changed behavior with respect to transcriptional regulatory activity in a cell type-dependent manner [40]. K189 is located in the protein region encoded by exon 5 that resides C-terminally to the KRAB domain. K286 is placed at the beginning of the C-terminal half of the protein and encoded by exon 7 (see Fig. 2a). In the previous experiments above we noted that the inclusion of the exon5–6 protein region contributed to the repressor function of the N-terminal ZNF746 protein fragment ZNF746/1–279. To analyze a potential role of sumoylation, we generated a mutant that replaced the SUMO target site lysine encoded in exon 5 with arginine (construct ZNF746a/1–279-K189R) and evaluated its repression potential in our heterologous reporter assay (Fig. 7b). In both, HeLa and HAP1 wildtype cells, the K189R replacement strongly decreased the repressive impact of the N-terminal segment to very poor (HeLa, 1.3fold) or completely abolished values (HAP1, 0.8fold). These results suggested a crucial potential role of sumoylation at residue K189 for the N-terminal half of ZNF746a to reach its full repression capacity in our cell model. However, we did not formally demonstrate that such sumoylation is taking place in our system. Our findings suggested that even with a truncated KRAB domain, the whole N-terminal protein configuration of isoform ZNF746a is able to impart transcriptional repression activity. We hypothesize that sumoylation at K189 may play a decisive role in fine-tuning transcriptional gene regulation of ZNF746.

### DUF3669 is a protein oligomerization domain

Inspection of first-neighbor protein networks in the human protein complex resource BioPlex2.0 (http://bioplex.hms.harvard.edu/ [51] indicated that ZNF746 and ZNF777 shared protein assemblies with other members of the DUF3669-KRAB-ZNF paralog group since both proteins were pulled down when either ZNF212 or ZNF398 was used as bait. Affinity purification with ZNF746 as bait followed by mass spectrometry identified complexes with cellular ZNF746, ZNF212, ZNF282 and ZNF783 [41]. Further, a yeast two-hybrid screen for interactions between various transcription factors recorded homo-oligomerization for ZNF212 [52]. Likely, ZNF746 and ZNF777 might co-exist in the same protein complex as well. Based on these data, we reasoned that DUF3669 might form an oligomerization interface for DUF3669-containing KRAB-ZNF proteins.

**Fig. 8:**
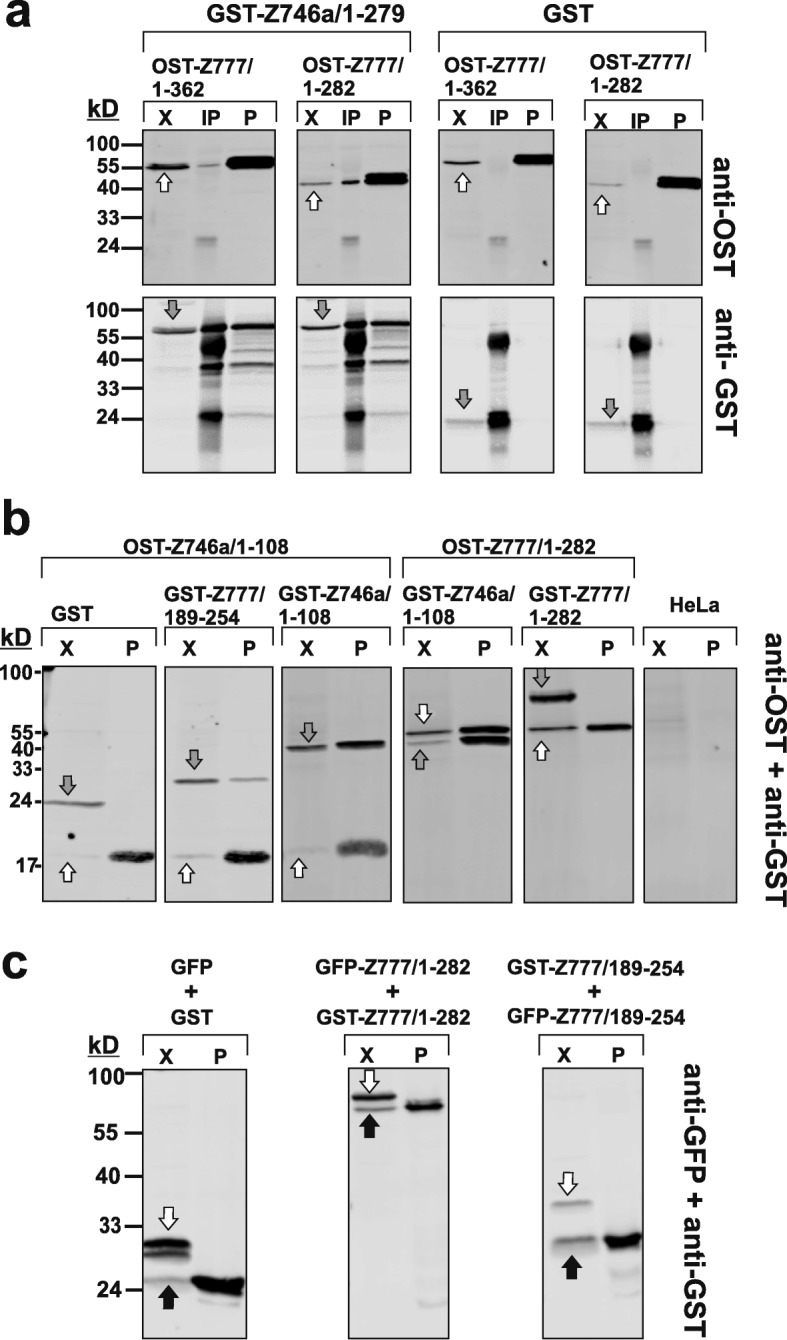
In vivo evaluation of homo- and hetero-oligomerization of DUF3669 domains of Z746 and Z777. **a** Analysis of oligomerization between N-terminal fragments of ZNF746 and ZNF777. HEK293 cells that stably express either GST or GST-Z746a/1–279 were transiently transfected with either Z777/1–362 or Z777/1–282 tagged with a One-Strep-Tag (OST). Proteins enriched by either immunoprecipitation with anti-GST rabbit polyclonal antibodies (lanes labeled “IP”) or by pull-down using Strep-Tactin via the OST (lanes “P”) were analyzed by Western blotting alongside an aliquot of the input extract (lanes “X”) with GST and OST antibodies in different color channels (upper and lower panels from the same blot) as indicated. **b** Narrowing down of the protein oligomerization domains and analysis of homo-oligomerization. HeLa cells were transfected with the indicated GST/GST-fusion and OST-fusion constructs and subjected to Strep-Tactin pulldown via the OST-tagged proteins. Western blots interrogated the input extracts and the enriched fractions (lanes labeled “X” and “P”, respectively) using anti-GST and anti-OST antibodies. Representations of the two-color channels are given here as black/white overlay images. **c** Alternative analysis of ZNF777 homo-oligomerization using HeLa cells transfected with GST/GST-fusion and GFP/GFP-fusion constructs. Enriched proteins from GST pulldown via glutathione beads (lanes “P”) were probed with anti-GST and anti-GFP antibodies. Images depict black/white representations of the two-color overlays. White block arrows point to GST bands, grey block arrows point to OST bands and black block arrows denote GFP bands

In order to show association and interrogate the domains responsible for possible interaction, we first performed co-immunoprecipitation and pull-down assays in human cell lines. A stably expressed GST fusion protein of the ZNF746a N-terminal segment made of DUF3669/KRAB/exon5–6 sequences (GST-ZNF746a/1–279) was able to co-immunoprecipitate the tagged DUF3669-KRAB segment of ZNF777 (OST-ZNF777/1–362) as well as a shorter version that only contained sequences encoded by the first exon including DUF3669 (OST-ZNF777/1–282; Fig. 8a). These results were corroborated by the reciprocal experiment: Enrichment of the respective ZNF777 protein part through its tag led as well to successful pulldown of the N-terminal ZNF746a protein segment (Fig. 8a).

We further narrowed down the protein segments responsible for association in the same complex and analyzed possible homo-oligomerization. Various constructs expressing tagged versions of segments containing only the DUF3669 of ZNF746 and ZNF777 and reciprocal pull-down through the respective tags indicated that the DUF3669 sequences contained in constructs ZNF746a/1–108 and ZNF777/189–254 were sufficient for association (Fig. 8b) evincing that DUF3669 serves as a hetero-oligomerization domain. Two versions of ZNF746 DUF3669 with different tags (OST- or GST-ZNF746a/1–108, respectively) were also able to associate in the same complex indicating a potential homo-oligomerization. In contrast, the ZNF777/1–282 protein portion did not form stable self-complexes when tested as OST- and GST tagged versions. This result was unexpected in light of the ZNF746 studies and prompted us to perform an alternative experiment by replacing the OST- with a GFP-tag. However, the observation stayed the same, there was no indication that the longer protein segments ZNF777/1–282 or the narrowed down DUF3669 region ZNF777/189–254 self-interacted (Fig. 8c). Thus, the DUF3669 of ZNF777 failed to form homo-oligomers.

**Fig. 9:**
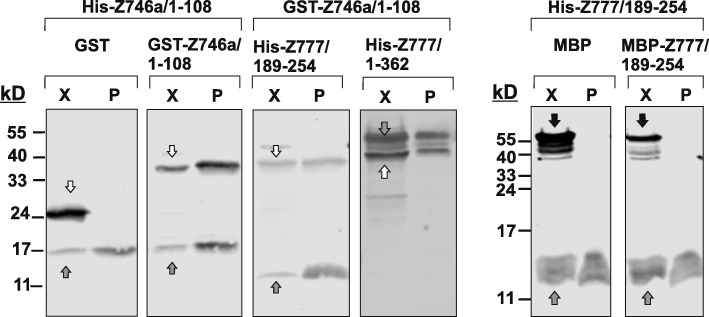
In vitro evaluation of homo- and hetero-dimerization of DUF3669-containing polypeptides of ZNF746 and ZNF777. Crude bacterial extracts containing the indicated recombinant proteins were mixed and used to pull-down His-tagged proteins. Western blots were probed with anti-His tag antibodies and either GST (the four panels to the left) or MBP (the two panels to the right) tag antibodies to visualize the proteins in the input extracts (lanes “X”) and to identify the enriched recombinant proteins. GST alone and MBP alone were used as negative controls. Images represent black/white overlays of the original two color channels. Grey arrows denote His-tagged proteins, white arrows to GST and GST fusion bands and black arrows to MBP and MBP fusion proteins

Protein association analysis in cell lines does not formally prove direct interaction and does not exclude the possibility of bridging molecules. This is why we performed pull-down experiments of recombinant proteins derived from bacterial extracts. Using GST and His-tagged protein segments we observed direct homo-oligomerization of ZNF746 DUF3669 (ZNF746a/1–108) and direct interaction, i.e. hetero-oligomerization, with the N-terminal segment as well as the narrowed down DUF3669 of ZNF777 (ZNF777/1–362 and ZNF777/189–254, respectively; Fig. 9). In contrast, but in agreement with the results in the human cell lines, the DUF3669 of ZNF777 (ZNF777/189–254) failed to self-associate.

These findings suggest that the DUF3669 plays a role in regulating the assembly of complexes with other proteins that contain the same domain. Despite high sequence homology, DUF3669 sequences of ZNF746 and ZNF777 were found to differ in that the former did but the latter did not confer homo-oligomerization.

## Discussion

In the present work, we characterized the unconventional amino-terminal configuration of ZNF746 and ZNF777 that consists of a DUF3669 and KRAB domain with respect to transcriptional repression potency and protein oligomerization behavior. Our data show that the DUF3669 portions of ZNF746 and of ZNF777 are sufficient to exert moderate repressor activity towards a strong viral promoter of the reporter gene cassette. At the same time, DUF3669 constitutes an oligomerization domain that mediates hetero-oligomerization between different DUF3669 protein members and, at least for ZNF746, homo-oligomerization as well. The high evolutionary conservation of DUF3669 sequences within an ortholog group and even between paralogs (Fig. 1) underscores the importance of these two functions for the two proteins. It is noteworthy, that a heterologous transfer of DUF3669 failed to further augment transcriptional silencing mediated by potent canonical KRAB-AB domains like the one from ZNF10. We favor as reason the requirement for a specific multi-domain organization including DUF3669, a certain non-canonical KRAB variant and adjacent sequences. Alternatively, a potent canonical KRAB-AB domain might be too dominant to observe any effects.

**Fig. 10:**
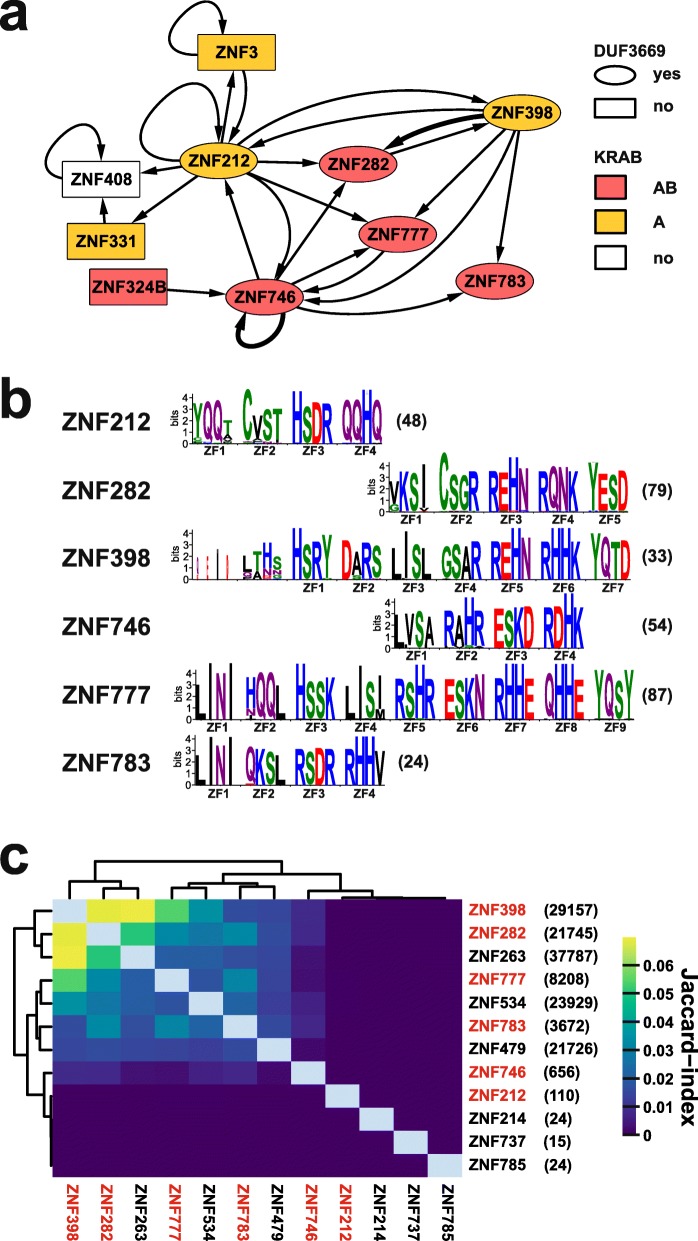
DUF3669-KRAB-ZNF protein complexes and DNA binding signatures **a**. DUF-KRAB-ZNF protein-protein interaction network taking into account only ZNF proteins found in protein complexes with any DUF3669-KRAB-ZNF protein. Arrows point from bait to prey. Line width reflects detection in one or two (thicker line) studies. Derived from data that were based on affinity-purification/mass spectrometry using tagged DUF3669-KRAB-ZNF proteins (Bioplex 2.0, [41, 51], a yeast two-hybrid system [52] and co-immunoprecipitation [53] and including our ZNF746/ZNF777 data). **b**. Zinc finger “DNA binding code” signatures for the DUF3669-KRAB-ZNF ortholog groups (see Methods). Each ortholog group depiction relied on the alignment of the individual signatures from all ortholog sequences (count given to the right). The zinc fingers (ZF) are numbered according to the human member. The horizontal placement of the different group signatures indicates some homologies between the whole paralog group. **c**. Heatmap describing the overlap between the genomic DNA binding sites based on the Jaccard statistic. We included available data for six DUF3669-KRAB-ZNF proteins and for the three ZNF proteins with the lowest and three with the highest number of binding sites in the dataset from [32] for comparison. The DUF3669-KRAB-ZNF proteins are labeled in red. The numbers of genomic binding sites are listed in brackets to the right of the protein name. See Methods for details and the included datasets

In our work here, we established a conserved 95 amino acid long DUF3669 as a protein interaction interface for oligomerization. Thus, one member or several DUF3669-containing proteins likely form higher-order molecular assemblies through this interface. Such complexes might be instrumental for function and open possibilities for context-dependent regulation if different DUF3669 members interact at distinct scenarios. Previous work already documented shared complexes of the DUF3669-KRAB-ZNF proteins, however, without pointing to the DUF3669 region as a potential protein-protein interaction interface [41, 51, 52]. Interestingly, protein assemblies sometimes also contained ZNF proteins without DUF3669. Evidence for the oligomerization property of DUF3669 was presented just recently, during the finalizing of our paper, using co-immunoprecipitation experiments of ZNF282 and ZNF398 [53]. We summarized the current knowledge about shared complexes of DUF3669-KRAB-ZNF proteins based on all this and our work here as a protein interaction network (Fig. 10a).

DUF3669-KRAB-ZNF proteins are likely addressing different gene sets, since the amino acids in their zinc finger motifs that have the major impact on DNA binding specificity are clearly specific and have been conserved during evolution (Fig. 10b). Genome-wide DNA binding profiles obtained in cell lines overexpressing tagged DUF3669-KRAB-ZNF proteins illustrated the differences in binding space but also showed significant overlap (Fig. 10c). First of all, the total count of the determined genomic binding sites differed vastly, from about 100 (ZNF212) up to about 30,000 (ZNF398). On the other hand, some similarity in the binding profiles is visible in the heatmap as well. ZNF282 and ZNF398 showed the highest intersection among the DUF3669-KRAB-ZNFs (Jaccard index 0.069). However, a ZNF protein without DUF3669 like ZNF263 shared a similar number of DNA binding sites with ZNF398 (Jaccard index 0.070). Earlier, more detailed analysis demonstrated proximity of promoter recruitment for ZNF282 and ZNF398 as well as ZNF282 and ZNF777 (see extended data Fig. 5 in [32]). In light of the use of transgenic cell lines these data might be biased towards representing the sole binding space of the respective overexpressed DUF3669-KRAB-ZNF protein. Conversely, DNA binding peaks describing genomic associations of potential hetero-oligomers of different DUF3669-KRAB-ZNF were likely underrepresented given the usual low endogenous expression of KRAB-ZNF proteins in cells. In the native cellular situation, oligomerization may offer increased stability or provide combinatorial binding site choices with respect to DNA binding. A low number of genomic binding sites might point to roles other than as primary transcription factor. Interestingly, interactome studies revealed that ZNF746 with only roughly 660 DNA binding sites shared complexes with RNA-binding proteins, among them ribosomal proteins and in particular six subunits of the large multicomponent eukaryotic initiation factor eIF3 complex [41, 53]. Both studies were conducted using the ZNF746a isoform with truncated KRAB-A. It is conceivable that isoform ZNF746b with full-length KRAB-A does not only show increased TRIM28 interaction and a boosted repressor activity like we observed here, but might display a different overall interactome as well. ZNF746 could therefore be subject to a scenario in which differential splicing leads to the expression of protein isoforms with differential transcriptional/translational regulatory potentials. It is important to bear this in mind when considering the role of ZNF746 in a certain biological context like in Parkinson’s disease [35]. Intriguingly, a very recent report illustrated isoform-dependent functions for ZNF398 on a posttranslational level: Only the isoform lacking amino-terminal sequences including DUF3669 and a major part of KRAB-A formed a complex with tumor suppressor TP53 and MDM2 and thus enhanced ubiquitinylation and subsequent degradation of TP53 [54].

Despite some KRAB-mediated inhibition of transcription and detectable interaction to TRIM28, the (full-length) KRAB domain of ZNF746 was clearly much less potent in repression and its extent of complex formation with TRIM28 lower than a bona fide KRAB domain like ZNF10-AB. Using mutational analysis in ZNF746 KRAB-A, we pinpointed the main reason for this difference to the lack of a glutamic acid residue in the functionally important “MLE” motif of canonical KRAB-A subdomains. Interestingly, not only was the missing glutamic acid residue evolutionary conserved in ZNF746 orthologs, but three out of the five other human DUF3669-KRAB-ZNF proteins share the same deficiency and display the same “MRG” or very similar “MKG” motifs throughout their ortholog groups (ZNF398, ZNF777, ZNF783; Additional file 3). In ZNF212 KRAB-A, the glutamic acid residue is offset by one position in the human ortholog and in some species as a motif “MES”, but in additional species displays “MKS”. The KRAB-A of the ZNF282 ortholog group is characterized by a motif “VKE” instead of “MLE” and thus differs from canonical KRAB-A as well (Additional file 3). The ZNF282 KRAB failed to transrepress and rather transactivated a reporter gene [46]. Low or missing transcriptional repression potency has thus been shown for the KRAB domains of ZNF746, ZNF777 (our data) and ZNF282. This is in agreement with our observation of weak or absent protein complex formation of ZNF746 and ZNF777 KRAB with TRIM28 which is the principal mediator of canonical KRAB activity. A recent publication also scored the DUF3669-KRAB-ZNF proteins ZNF212, ZNF398, ZNF746 and ZNF777 as “weak TRIM28 binders”, even despite the use of crosslinking before affinity enrichment from cellular extracts [53]. Altogether, the sequence conservation of non-canonical KRAB domains in DUF3669-KRAB-ZNF proteins during evolution argues that mechanisms other than recruitment of TRIM28, SETDB1 and associated proteins and activities are intrinsic and important for this transcription factor subgroup when considering the role of their KRAB region.

In our heterologous reporter system here, removal of a known SUMO acceptor lysine in the ZNF746a protein region encoded by exon 5 abolished the repressive activity of the whole N-terminal DUF3669/KRAB/exon5–6 protein fragment. This result is in agreement with previous work that employed a different heterologous reporter system with a VP16 co-activator and full-length ZNF746 of the type with truncated KRAB-A as a test protein [40]. However, the complexity of the situation was unraveled by the same authors when studying the transcriptional activity of ZNF746 towards a reporter containing promotor sequences of the authentic target gene PGC-1alpha. They found cell type-dependent opposite effects of the SUMO mutants, either canceling of wildtype ZNF746’s repression activity like in our situation or a transactivating impact compared to controls. Clearly, these confusing data did not give rise to a generalized model describing the effect of sumoylation on transcriptional modulation by ZNF746. ZNF282, ZNF398 and ZNF783 also contain C-terminally to their DUF3669 and KRAB domain experimentally determined sumoylation sites [55]. A sumoylation-defective mutant of ZFP282, the mouse ortholog of ZNF282, reduced its transactivating capacity towards a reporter construct under control of an estrogen response element. This effect was accompanied by a destabilization of the complex that ZFP282 forms with estrogen receptor alpha and the protein “calcium binding and coiled-coil domain 1” [56]. Analysis of the amino acid sequence of human ZNF777 revealed that K370 is located within a perfectly matching consensus sequence for SUMO-conjugation (ψKxE, where ψ represents a large hydrophobic amino acid, K is the lysine conjugated to SUMO, x is any amino acid, and E is a glutamic acid) [57]. Regulation by sumoylation may therefore be a common theme for DUF3669-KRAB-ZNF proteins and should be studied in detail in the future. Sumoylation has variegated roles in nuclear biology and in the modulation of chromatin structure and function (for review see [58]). The posttranslational conjugation of SUMO to a protein can influence protein stability, confer a novel protein-protein interaction interface for assembly of large complexes and change the intracellular distribution of a protein. In light of transcription factors like the DUF3669-KRAB-ZNF proteins, a SUMO moiety might guide the assembly of complexes that modulate transcription in a context-dependent manner.

We employed N-terminal protein segments for our analysis of DUF3669 oligomerization in human and bacterial cells after ectopic expression. The above-cited work relied on overexpressed tagged proteins as well. In order to better describe the native situation, including stoichiometry of oligomerization, further work will be required to investigate the endogenous, full-length proteins at their bona-fide target genes.

## Conclusions

Our study revealed the added functionality for transcriptional modulation and oligomerization that DUF3669 provided for a subset of KRAB-ZNF transcription factors. Oligomerization and a weak repressor activity were both intrinsic properties of DUF3669 alone. A DUF3669 substantially contributed to the repressor potency in its original N-terminal domain context that includes a non-canonical KRAB domain and an adjacent region that can be subject to sumoylation. DUF3669 domains are likely parts of N-terminal protein configurations in their authentic proteins where they are functionally connected to and most likely add novel functionalities and/or properties to non-canonical KRAB domains that display weak or no repressor activity mediated via TRIM28.

## Methods

### Construction of expression vectors

The DNA sequence for full-length ZNF746a was purchased from BioCat GmbH/Heidelberg (BC068505-seq-TCHS1003-GVO-TRI). The DNA sequences encoding individual protein regions of ZNF746 and ZNF777 were synthesized by RT-PCR using High Capacity cDNA Reverse Transcription Kit with RNA inhibitors (Applied Biosystems, #4374966) and total RNA derived from human fetal brain (Clontech #64094-, 1 mg/ml conc.; pooled from 24 male/female Caucasians) and human testis (Clontech #64101–1, 1 mg/ml; pooled from 45 Caucasians age 19–64) unless otherwise stated. The PCR primers contained flanking restriction sites for later subcloning and are listed in Additional file 4. PCR products were cloned blunt-end into cloning vector pBluescript KS (Stratagene) and Sanger sequencing was used for verification. The desired expression cassettes were inserted as *Xho*I/*Sal*I fragments into the pM3 vector [59] restricted with *Sal*I to generate a coding sequence for a fusion protein with the DNA binding domain of the yeast transcription factor GAL4 at its N-terminus. Correspondingly, eukaryotic expression vectors encoding fusion proteins with glutathione-S-transferase from *Schistosoma japonicum* (GST) were generated by insertion of the *XhoI*/*Sal*I fragments into pN2-GST [48] digested with *Sal*I. The sequence encoding full-length ZNF777 (amino acids 1–831) was assembled in the pM3-ZNF777/1–362 construct by inserting the respective fragment from a commercial truncated ZNF777 plasmid (BioCat GmbH/Heidelberg; Cat# BC023985-seq-TCHS1003-GVO-TRI) into the *Sac*I site. The coding sequences of mutant protein regions (Z746b/109–188_R141L, Z746b/109–188_G142E, Z746b/109–188_RG/LE, and Z746a/94–279-K189R) with flanking *Xho*I/*Sal*I overhangs were purchased as in-vitro-synthesized DNA fragments that were cloned into the *EcoR*V site of the pUC57 vector (Eurogentec). *Xho*I-*Sal*I fragments were subcloned into pExpr-IBA105 (IBA GmbH #2–1905-000) digested with *Xho*I to generate a coding sequence of N-terminal One Strep-tagged proteins. To construct a coding sequence of a chimeric fusion protein between DUF3669 and KRAB domain of ZNF10, the coding sequences of DUF3669 domains of both ZNF777 and ZNF746 without stop codon (Z746a/1–108 and Z777/189–254, respectively) were first cloned as *Xho*I/*Sal*I fragment into pM3/*Sal*I. Then the resulting plasmids were linearized with *Sal*I and the coding sequence of the KRAB domain of ZNF10 was inserted into this site as *Xho*I/*Sal*I fragment. *EcoR*I/*Sal*I DNA fragments for Z777/189–254 and Z777/1–282 were subcloned into pEGFP-C1 (Clontech #6081–1) restricted with *EcoR*I/*Sal*I to generate a coding sequence of N-terminal GFP fusions. To clone GST and GST-Z746a/1–279 into a pcDNA5-FRT-TO expression vector (Invitrogen # V6520–20), the respective pN2-GST plasmid was restricted with *Hind*III/*Sal*I restriction enzymes, and then the resulting fragments were inserted into pcDNA5-FRT-TO plasmid restricted with *Hind*III/*Xho*I restriction enzymes. For prokaryotic expression, the GST-Z746a/1–108 encoding *Bgl*II-*Sal*I cassette was subcloned into bacterial expression vector pRSFDuet1 (Merck/Novagen #71341–3) restricted with *Bgl*II-*Xho*I. To construct a coding sequence for maltose-binding protein fusions (MBP), the *Sac*I-*Sal*I fragments were subcloned into pMALc2x (New England Biolabs) restricted with *Sac*I-*Sal*I. To generate 5xHis tagged proteins, *Xho*I/*Sal*I fragments were subcloned into pRSET-B (Invitrogen # V351–20) restricted with *Xho*I. The faithful expression of the different constructs was monitored by Western blotting after transfection in HeLa or HAP1 cells or after transformation of *E. coli* bacteria (Additional file 5 and Additional file 6).

### Bacterial expression and recombinant protein pull-down assay

The desired recombinant GST-, MBP- and His-tagged proteins were expressed in *E. coli* BL21 (DE3) bacteria (Amsbio C700200) after transformation with the respective prokaryotic expression vector. Firstly, a single colony was picked from an agar plate and incubated in 2 ml of glucose-rich growth medium (RMG; for 1 l: 10 g tryptone, 5 g yeast extract, 5 g NaCl, 2 g glucose) supplemented with the appropriate antibiotic at 37 °C and 200 rpm overnight. Next day, the cultures were expanded by inoculating 50 μl of the overnight culture into 5 ml of fresh RMG medium under the same conditions until reaching OD_600_ nm ≈ 0.5. The bacteria were pelleted by centrifuging for 10 min at 2.777 x *g* and 4 °C; washed with 5 ml PBS and re-suspended in 5 ml SB-medium (component A for 1 L: 12 g peptone from casein, 24 g yeast extract, 4 ml glycerol, component B for 1 L: 340 mM KH2PO4, 1.44 M K2HPO4; ready-to-use medium: 950 ml component A + 50 ml B) with the appropriate antibiotic. Expression was induced by adding 0.1 mM isopropyl β-D-1-thiogalactopyranoside to the SB bacterial suspension for 4 h at 37 °C. The bacteria were then pelleted by centrifuging for 10 min at 9520 x *g* and 4 °C, the pellets were washed and re-suspended with 1.5 ml extract buffer; 50 mM TRIS/HCl (pH:8), 300 mM NaCl, protease inhibitors (cOmplete ULTRA Mini EDTA free, Roche), 0.1% bovine serum albumin, 20 mM Imidazole and 0.05% Tween20, and 20% glycerol. The cells were lysed by four 15-s sonication cycles. The lysates were cleared by centrifugation for 30 min at 16060 x *g* and 4 °C. The expression levels and quality of the different recombinant proteins in these crude extracts were analyzed by Western blotting and served as input for pull-down assays. An appropriate aliquot (70–200 μl) of a His-tagged recombinant protein extract was combined in-vitro with an aliquot of another extract containing GST- and MBP fusion proteins (40–75 μl) in different combinations and mixed with 20 μl Ni-NTA magnetic bead suspension (Qiagen, Cat. 36,111) in extract buffer. After overnight incubation at 4 °C on a rotating mixer, the beads were washed 5 × 5 min with wash buffer; 50 mM TRIS/HCl (pH:8), 300 mM NaCl supplemented with 0.1% bovine serum albumin, 20 mM Imidazole and 0.05% Tween20 followed by washing for 3 × 5 min with the same buffer without albumin. Elution was done with 40 μl per tube of 250 mM imidazole dissolved in 50 mM TRIS/HCl (pH:8), 300 mM NaCl.

### Cell culture and transfection

Cell culture was done under standard conditions (humidified incubator at 37 °C and 5% CO2). All media were supplemented with 10% heat-inactivated fetal calf serum (FCS, Biochrom FBS Superior S0615), 45 units/ml penicillin and 45 μg/ml streptomycin (Gibco #15070–063). Hela cells (source: German Cancer Research Center, Heidelberg) and Flp-In™ T-REx™ 293 cells (Thermo Fisher Scientific R78007) were grown in DMEM (Gibco #31966–021), whereas HAP1 cells (Horizon Genomics; wildtype C631, TRIM28 knockout HZGHC000293c001, and SETDB1 knockout HZGHC001331c001) were grown in IMDM medium (Gibco #12440–053). Flp-In™ T-REx™ 293 cells were in addition kept in the presence of 100 μg/ml Zeocin (Invitrogen #64–0509) and 15 μg/ml Blasticidin (PAA Laboratories GmbH # P11–017). All cell lines were passaged using trypsin/EDTA. We used lipid-based commercial reagents for all transfections according to the manufacturer’s instructions. All transfections were conducted at about 70–90% cell confluency. For dual luciferase assay; HAP1 wild type and knockout cells were transfected using TurboFectin 8.0 reagent (OriGene, #TF81001), whereas HeLa cells were transfected using X-tremeGENE 9 (Roche, #06365787001). For immunoprecipitation and affinity pull-down assays, HeLa cells and Flp-In™ T-REx™ 293 cells were transfected with FuGENE® HD (Promega, #E2311). Transfection complexes were formed at 3 μl transfection reagent per 1 μg DNA in Opti-MEM® I medium (Gibco #51985026) during a 15–20 min incubation at room temperature and added to the cells dropwise.

For the generation of stable cell lines expressing GST alone or GST-Z746a/1–279, the parent cells (Flp-In T-rex 293) were co-transfected with either pcDNA5-FRT-TO-GST or pcDNA5-FRT-TO-TNF746a/1–279 (confers Hygromycin B resistance and expresses respective GST protein) together with plasmid pOG44 (Thermo Fisher #V600520; expresses Flp recombinase) in medium without Zeocin. Two days after transfection, selective pressure (250 μg/ml Hygromycin B) was applied. Visible colonies were picked about 2 weeks later, expanded and screened for expression of the desired protein by indirect immunofluorescence and Western blotting (Additional file 7). Expression of the transgene was induced by 2 μg/ml tetracycline for 24 h before analysis.

### Protein complex analysis in eukaryotic cells

Testing for the presence of the presumable protein interaction partners in the same protein complex was done using co-immunoprecipitation or affinity pull-down assays based on ligands for the tags of the interrogated proteins after transfection of respective constructs. The scale for co-immunoprecipitation was usually 0.5–1 million cells per 6-cm dish grown for 2 days, transfected with a total amount of 5 μg expression vector(s) and harvested 24 h post-transfection. Affinity pull-down assays used the same scale or single wells of 6-well plates transfected with a total of 2 μg DNA. After a quick rinse in cold PBS, cells were lysed in ice-cold TST buffer (20 mM TRIS/HCl pH 7.5; 60 mM KCl, 15 mM NaCl, 10 mM MgCl2, 1 mM CaCl2, 250 mM Sucrose, 0.5% Triton X-100) supplemented with protease inhibitors (cOmplete ULTRA Mini EDTA-free, Roche), 1 mM DTT and phosphatase inhibitors (1 mM Na3VO4, 50 mM NaF, 40 mM beta-glycerophosphate) as described previously [48]. For co-immunoprecipitation, protein G Mag-Sepharose (MagGS) Beads (GE Healthcare) were loaded with monoclonal anti TRIM28 (mAb 1Tb1A9, [60]; a kind gift of Pierre Chambon and Régine Losson; Illkirch, France) or polyclonal antibodies against GST (Santa Cruz Biotechnology, sc-459). One-Strep-tag tagged proteins were pulled-down from TST extracts using Streptactin-Superflow (IBA GmbH, #2–1206-010). For each sample, we used 25 μl of packed beads (50 μl suspension). Glutathione magnetic agarose beads (Pierce™; #78601; 12.5 μl packed beads in 50 μl TST buffer per sample) were used to pull-down GST fusion proteins. TST cell extracts were in each case mixed with the beads and incubated on a rotating shaker overnight at 4 °C. Beads were either collected on a magnetic stand or by centrifugation at 3427x *g*. The beads were washed three times with ice-cold TST buffer supplemented with 0.5% Triton X-100 and finally rinsed with TST buffer without Triton-X-100. Elution was done with SDS sample buffer (in the case of co-immunoprecipitation), 3 mM D(+) biotin (Carl Roth GmbH #3822.1; for enrichments based on One-Strep tag) or 50 mM reduced glutathione (Sigma-Aldrich #G4251-1G when using glutathione beads). Eluates and cell lysate aliquots of each sample were then subjected to Western blotting to probe for co-enriched endogenous TRIM28 protein or tagged potential interaction partners encoded by co-transfected vectors.

### Dual luciferase reporter assay

Cells were co-transfected with:750 ng effector plasmid (pM3-based; see above), 10 ng *Renilla* luciferase reporter plasmid (hRLuc/Tk; Promega # E692A, and 250 ng *firefly* luciferase reporter plasmid that contains five GAL4 DNA binding site upstream of a strong SV40 promoter (pGl3control-5’Gal4 [48];). Cells were subjected 24-h post-transfection to a dual luciferase assay (Promega) following the manufacturer’s protocol and using a Berthold single channel luminometer (Lumat LB9501). *Firefly* luciferase activities were normalized with respective *Renilla* luciferase activities for each sample. The luciferase activity in the presence of the effector plasmid expressing the GAL4 DNA binding domain alone represents the reference transcriptional capacity. Fold repression was calculated experiment-wise by dividing the normalized luciferase activity of that reference through normalized luciferase activities of the sample with the respective test effector plasmid. Note that identical results for certain constructs are presented in more than one figure for ease of comparison.

### Western blotting

Western blotting was essentially performed as described [48] using Lämmli-type denaturing sodium dodecyl sulfate-polyacrylamide gels and semi-dry electroblotting onto low-fluorescence PVDF membranes (Immobilon®-FL, Millipore IPFL00010). Immunostaining was done with the following primary antibodies: Rabbit antibodies against GST (glutathione-S-transferase from *Schistosoma japonicum*; used at 0.2 μg/ml; Santa Cruz Biotechnology, sc-459), against MBP (maltose-binding protein from *E. coli*; used at 0.1 μg/ml Santa Cruz Biotechnology, sc-808) as well as monoclonal antibodies against One-Strep-tag (at 0.1 μg/ml; IBA GmbH, #IBA2–1507-01), against TRIM28/KRIP-1 (at 0.25 μg/ml; BD Biosciences #610681), against Penta-His (0.1 μg/ml; Qiagen #34660), and against GFP (green fluorescent protein from *Aequorea victoria*; used at 0.4 μg/ml; Roche #11814460001). Primary antibodies were detected by fluorescently-tagged secondary antibodies against rabbit or mouse IgG (LI-COR # 926–32,211, # 926–32,220, 0.1 μg/ml for each) and signals were acquired using an LI-COR Odyssey® CLx imager. Individual image panels presented in figures were always subjected channel-wise “in one piece” simultaneously to any contrast or brightness adjustment within the LI-COR Image Studio Lite 5.2 software before export as black-and-white image and any cropping. Even when present side-by-side on the same gel/blot, lanes were grouped into panels for individual constructs for clearer presentation and easier comprehension. Images were also cropped to only show the molecular weight regions that are informative for our proteins on interest.

### Bioinformatic analysis

We compiled all orthologs of the seven human DUF3669-containing proteins from the TreeFam database release 9 (www.treefam.org). The DUF3669-containing proteins were specified by an Ensembl database identifier and were categorized into the seven orthologous protein groups of ZNF212, ZNF282, ZNF398, ZNF746, ZNF767, ZNF777 and ZNF783. For each identified protein we extracted the DUF3669-containing polypeptides including all other residues encoded by the same single exon from Ensembl Release 95 (January 2019). We then employed multiple alignments (ClustalW; https://www.genome.jp/tools-bin/clustalw) and profile Hidden Markov Model (HMM) software (HMMbuild; HMMER 2.3.1 [61];) to generate consensus sequence matrices for DUF3669. In addition, we searched for more DUF3669-containing proteins in the animal phyla using the protein BLAST servers of NCBI and Uniprot 2019_02. Ortholog group assignments took into account sequence homology and the results of reciprocal BLASTp searches in the respective species. Sequences that could not be unequivocally attributed to a human ortholog group were put into the group “unassigned”. The compiled proteins are listed in Additional file 1. The HMM matrix for DUF3669 and matrices for KRAB-A and KRAB-B [1] were used to retrieve the respective domains which were then aligned by ClustalW. Interaction between a C2H2 zinc finger and specific bases of DNA has been proposed to rely on key amino acid residues at position − 1, 2, 3 and 6 of the alpha-helix of a finger [62]. We extracted this 4-letter “zinc finger code” for all zinc fingers of a ZNF protein and aligned the resulting signatures for each human DUF3669-KRAB-ZNF ortholog group using ClustalW. Positions with information in less than 10% of the sequences were removed from the alignment for clarity. The various alignments described above were used to generate sequence logo representations (WebLogo 3.7.4, http://weblogo.threeplusone.com/create.cgi) [63]. Protein-protein networks were visualized with Cytoscape 3.7.1 [64]. Overlapping genomic binding sites of different ZNF proteins were evaluated using the Jaccard statistic (at least 50% overlap) of the BEDTools v2.29 suite (https://bedtools.readthedocs.io/en/latest/content/tools/jaccard.html [65];). The bed files originated from data of [32, 53] and from ENCODE (from GSE78099 set: GSM2466645, GSM2466498, GSM2466660, GSM2466515, GSM2466497, GSM2466659, GSM2466545, GSM2466654, GSM2466568, GSM2466586 and GSM2466508; ENCFF266KSD, ENCFF612GGC, ENCFF058JCF, GSM3402720, and the ZNF263 data extracted from ENCODE-TFBS at https://hgdownload.cse.ucsc.edu/goldenpath/hg19/encodeDCC/wgEncodeRegTfbsClustered/). The heatmap was constructed using the R programming environment (www.r-project.org) and clustering with Euclidean distance, average linkage.

## Supplementary information

**Additional file 1.** Compilation of DUF3669-KRAB-ZNF proteins from amniotes. Individual sheets for the ortholog groups based on the human members, an “unassigned” group and a table with a group count.

**Additional file 2.** Negative control luciferase reporter assays in HeLa cells and the HAP1 cell system (wildtype, TRIM28 knockout and SETDB1 knockout cells, respectively) using Gal4 fusion constructs with segments of human PRDM9 (P9/24–97, covering the KRAB-like domain; P9/24–363, covering the extended N-terminal KRAB-SSXRD-PRSET part; see reference [48]). Assay procedure as described in legends of Figs. 3 and 4. Bar plots represent normalized mean repression factor values ± STDEV of four (HeLa) or six (HAP1 system) biological replicates from two (HeLa) or three (HAP1) independent experiments relative to experiment-specific values for the Gal4-DBD alone.

**Additional file 3.** Amino acid sequence logos for KRAB-A boxes in the DUF3669-KRAB-ZNF ortholog groups along with the sequences of the respective human member (“hs”) below each logo. The counts to the right indicate the number of ortholog sequences that are represented in each logo. For comparison, the canonical KRAB-A of human ZNF10 (RefSeq NP_056209.2) is depicted on top. HMMER scores against a human HMM matrix of KRAB-A [1] are given to the right of each individual sequence. Note, that the occurrence of orthologs with truncated N-terminus is visible in the ZNF746 logo and the KRAB-A sequences of both isoforms are given. The highlighted amino acids represent the position of the “MLE” motif in canonical KRAB-A.

**Additional file 4.** List of PCR primers.

**Additional file 5.** Verification of expression and expected size of the Gal4 fusion proteins encoded by the constructed pM3 expression vectors using Western blotting in HeLa cells (a, b, c, d, e, f) and HAP1 wild type cells (g - i). Cell extracts made 24 h post-transfection with 1 x SDS sample buffer were probed with rabbit polyclonal antibodies against GAL4 (upper panels; Santa Cruz Biotechnology sc-577 at 0.2 μg/ml) and monoclonal antibodies against endogenous GAPDH (lower panels; Abcam ab8245 at 0.1 μg/ml). Non-transfected HeLa cell lysates are used as negative controls. Black block arrows point to bands of expected size when more than one protein species is visible; * indicates bands due to cross-reactivity of the antibodies. Panels a-i cover all Gal4 fusion protein constructs used in the manuscript.

**Additional file 6 **In vitro expression of DUF3669-containing polypeptides derived from ZNF746 and ZNF777. SoluBL21 (DE3) *E. coli* were transformed with 10 ng of the indicated prokaryotic constructs. The protein expression was induced by adding 0.1 mM IPTG to the bacterial suspensions at OD600 = 0.4–0.6. Bacterial cell pellets were lysed with 1 x SDS sample buffer (ni = non-induced bacteria, 25 μl total extract; i = bacteria after 4 h induction with IPTG, 25 μl). Soluble crude fractions of recombinant proteins were obtained from induced bacteria that were washed with 1x ice-cooled PBS, re-suspended in lysis buffer and lysed by sonication. Different amounts of bacterial lysates were loaded to the SDS-polyacrylamide gels (L1: 4 μl, L2:8μl, L3:20μl). Extracts were subjected to Western blotting and the membranes probed with polyclonal anti-GST and monoclonal anti-His tag (a, two-color results shown as one black/white overlay representation), polyclonal anti-MBP (b) or monoclonal anti-His tag (c) antibodies.

**Additional file 7.** Analysis of stable HEK293 cell lines expressing GST alone or GST-Z746a/1–279 Western blot analysis of total protein extracts from six clones that survived under the selection of hygromycin B (2 express GST and 4 express GST-Z746a/1–279) alongside extracts from parent cell line Flp-in T-REx-239 cells (negative control) and transiently transfected parent cells expressing GST or GST-Z746a/1–279 (positive controls). Extracts made with 1x SDS sample buffer after a 24-h induction of expression with 2 μg/ml tetracycline. The blot was probed with anti-GST (depicted in the upper part) and anti-GAPDH (lower part) antibodies. Protein bands of the expected size are indicated by arrows. * indicates unspecific bands recognized by the antibodies.

## Data Availability

All data generated or analyzed during this study are included in this published article [and its supplementary information files]. Exceptions are several large-scale datasets that we used but were generated by others. Those were referred to by accession numbers of the Gene Expression Omnibus or ENCODE.

## Abbreviations

DUF: Domain of unknown function
GFP: Green Fluorescent Protein
GST: Glutathione S Transferase
KO: Knockout
KRAB: Krüppel-associated box
MBP: Maltose- binding protein
OST: One Strep Tag
PGC-1α: Peroxisome Proliferator-Activated Receptor gamma (PPARγ) coactivator-1a
SETDB1: SET Domain Bifurcated Histone Lysine Methyltransferase 1
TRIM28: Tripartite Motif Containing 28
ZNF: Zinc finger
