# Supplementary figures and images for: DUF3669, a “domain of unknown function” within ZNF746 and ZNF777, oligomerizes and contributes to transcriptional repression
Source: BMC Mol Cell Biol. 2019 Dec 19;20:60. doi: 10.1186/s12860-019-0243-y (PMC6923878; doi:10.1186/s12860-019-0243-y)

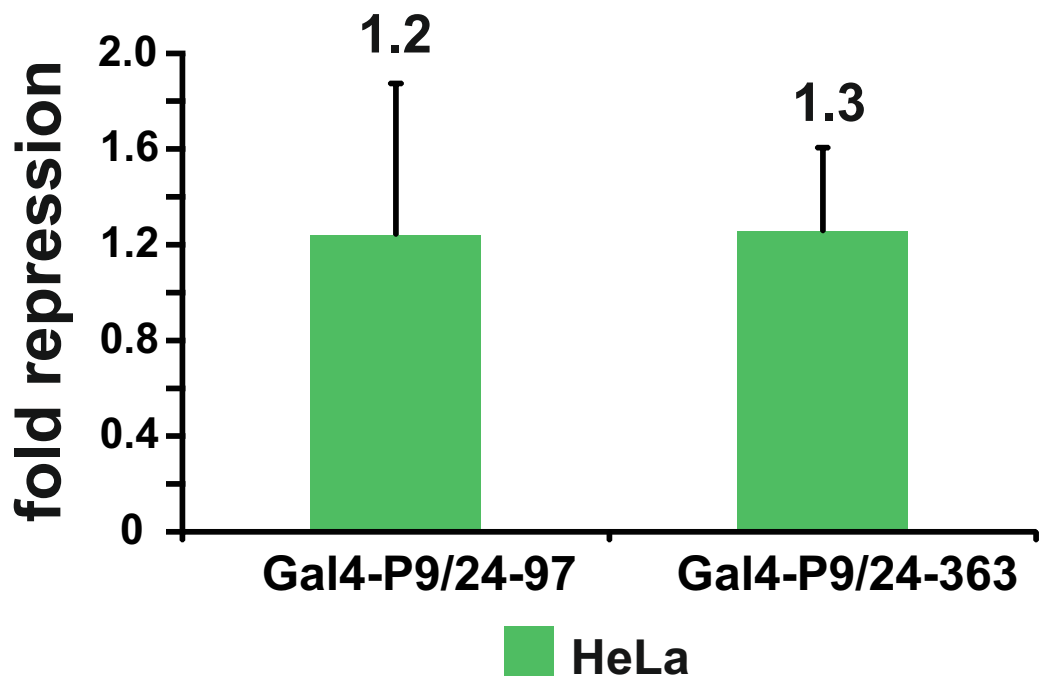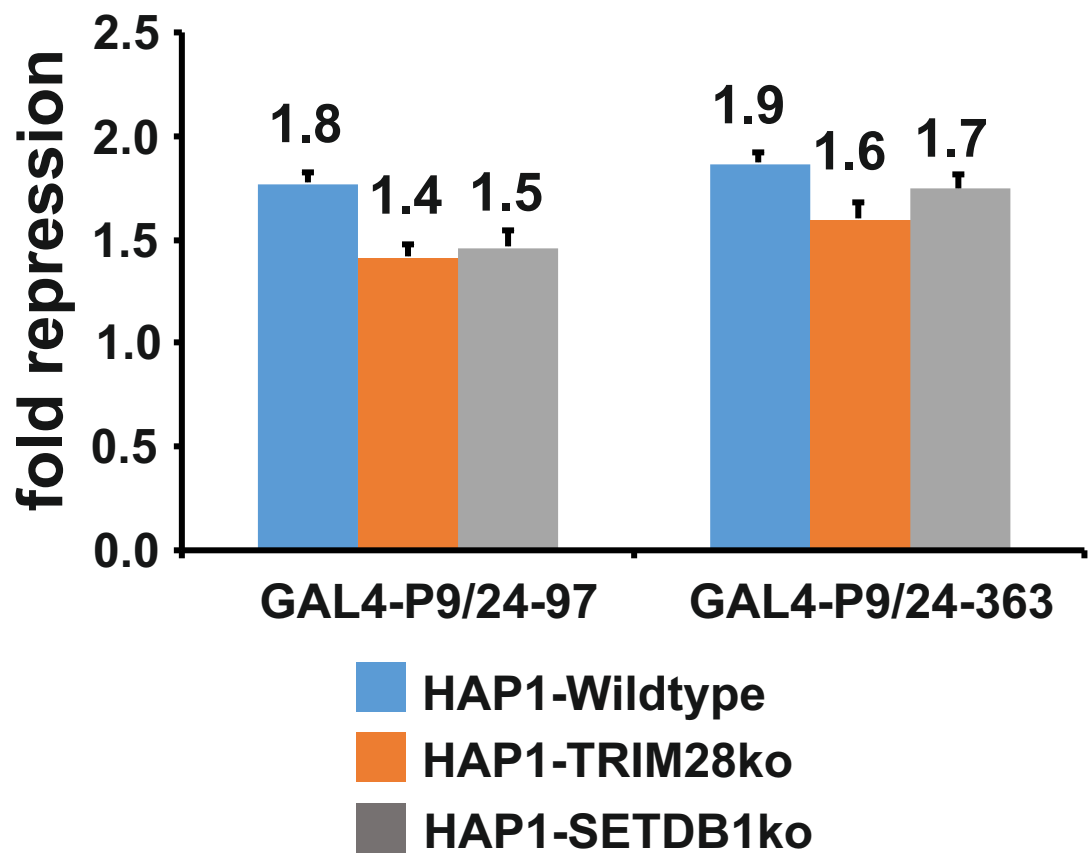

Supplement: Supplementary file 2 — Additional file 2. Negative control luciferase reporter assays in HeLa cells and the HAP1 cell system (wildtype, TRIM28 knockout and SETDB1 knockout cells, respectively) using Gal4 fusion constructs with segments of human PRDM9 (P9/24–97, covering the KRAB-like domain; P9/24–363, covering the extended N-terminal KRAB-SSXRD-PRSET part; see reference [48]). Assay procedure as described in legends of Figs. 3 and 4. Bar plots represent normalized mean repression factor values ± STDEV of four (HeLa) or six (HAP1 system) biological replicates from two (HeLa) or three (HAP1) independent experiments relative to experiment-specific values for the Gal4-DBD alone. [file 12860_2019_243_MOESM2_ESM.pdf]

# KRAB-A

## hsZNF10

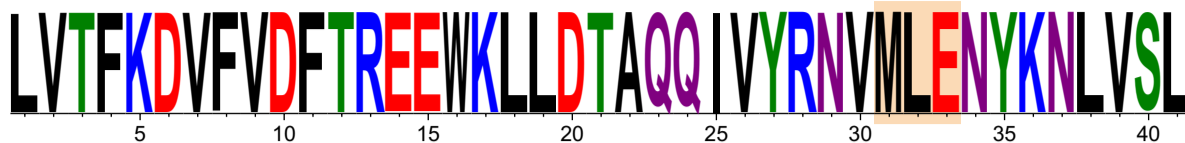

**2.1E-26**

# ZNF212

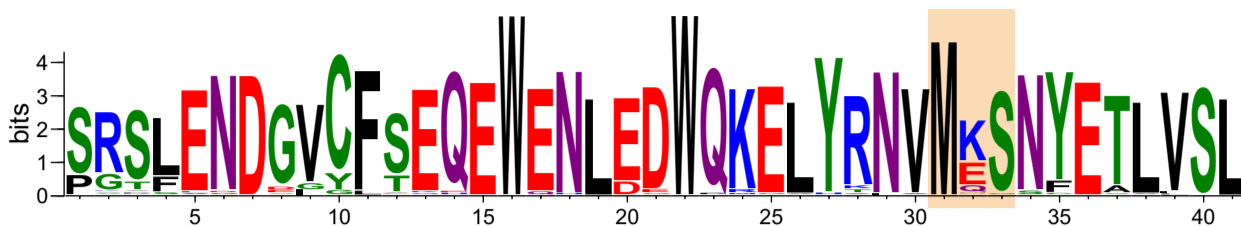

49

## hs

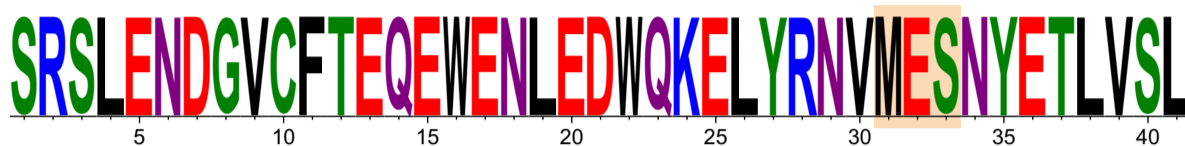

**1.1E-16**

# ZNF282

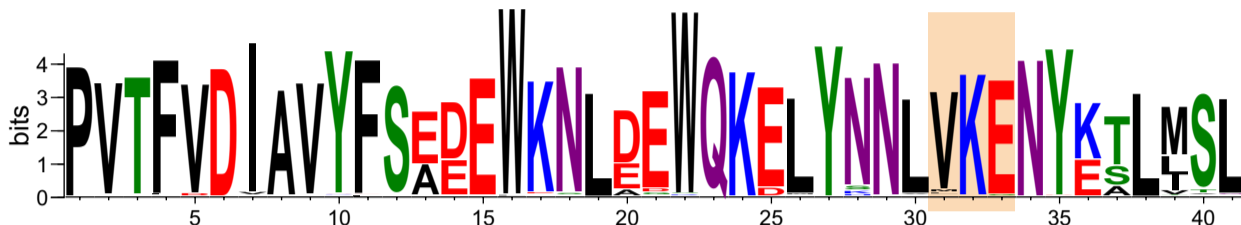

99

# hs

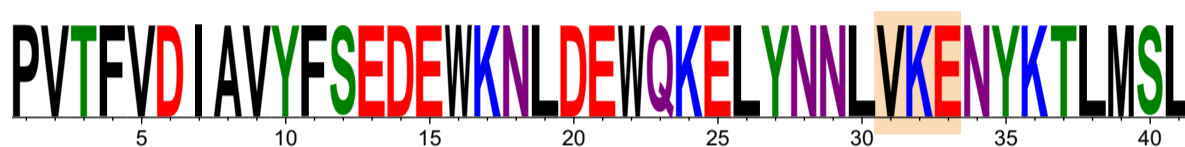

4.1E-21

# ZNF398

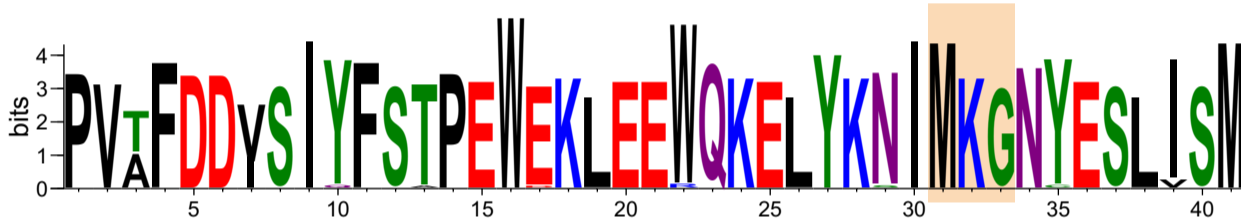

33

# hs

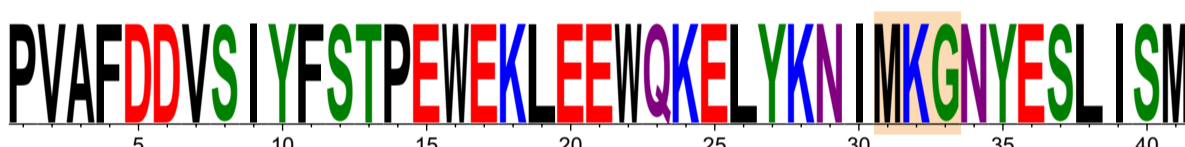

**4.7E-20**

# ZNF746

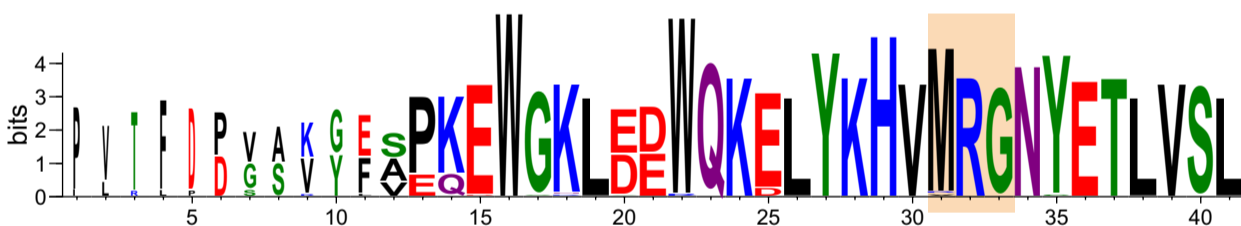

58

## hsZNF746a

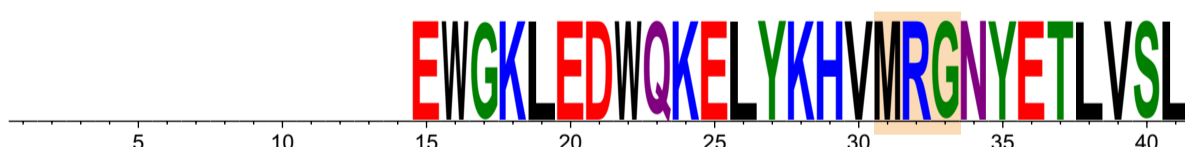

1.2E-07

## hsZNF746b

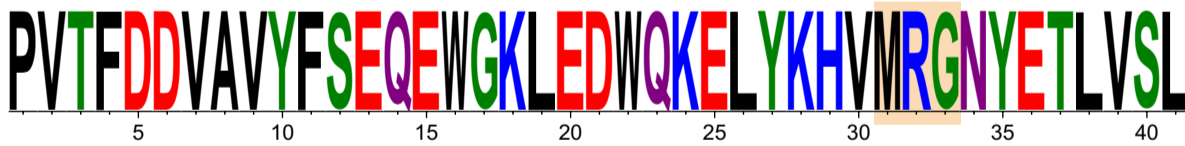

**7.5E-24**

# ZNF777

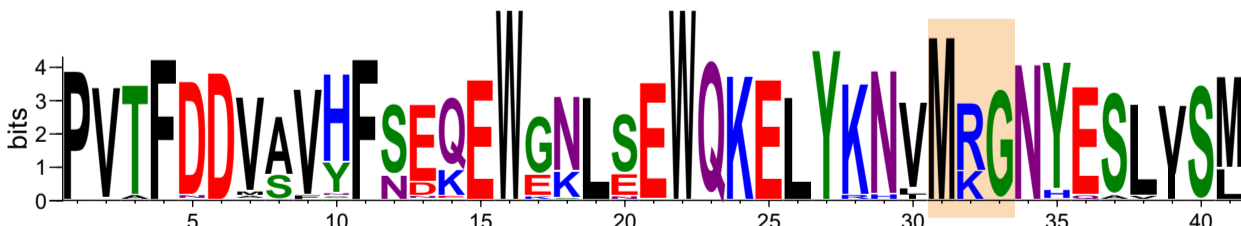

90

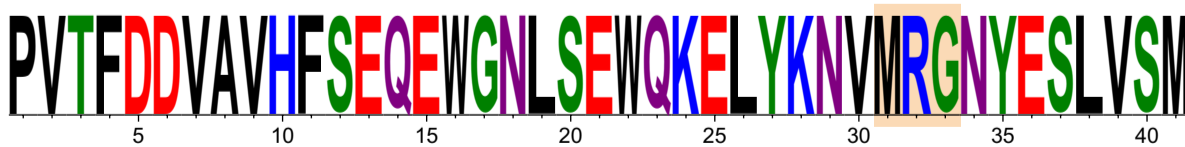

**2.4.10E-23**

# ZNF783

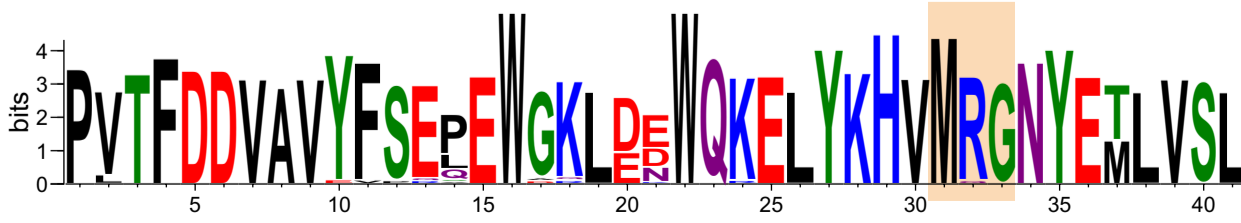

33

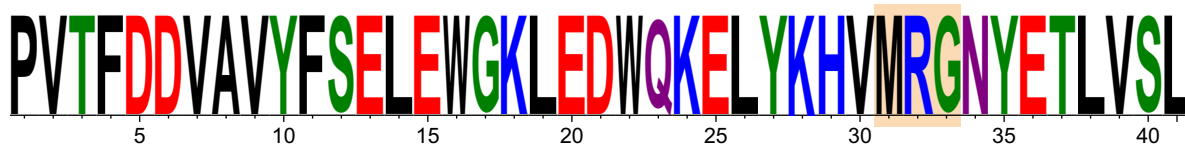

**4.3E-23**

Supplement: Supplementary file 3 — Additional file 3. Amino acid sequence logos for KRAB-A boxes in the DUF3669-KRAB-ZNF ortholog groups along with the sequences of the respective human member (“hs”) below each logo. The counts to the right indicate the number of ortholog sequences that are represented in each logo. For comparison, the canonical KRAB-A of human ZNF10 (RefSeq NP_056209.2) is depicted on top. HMMER scores against a human HMM matrix of KRAB-A [1] are given to the right of each individual sequence. Note, that the occurrence of orthologs with truncated N-terminus is visible in the ZNF746 logo and the KRAB-A sequences of both isoforms are given. The highlighted amino acids represent the position of the “MLE” motif in canonical KRAB-A. [file 12860_2019_243_MOESM3_ESM.pdf]

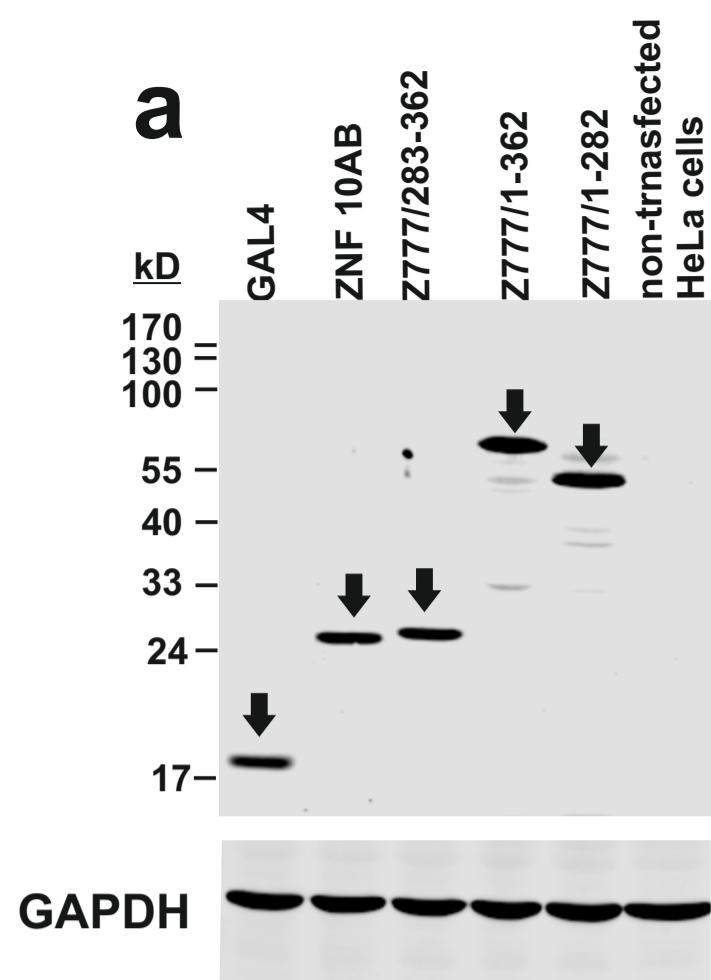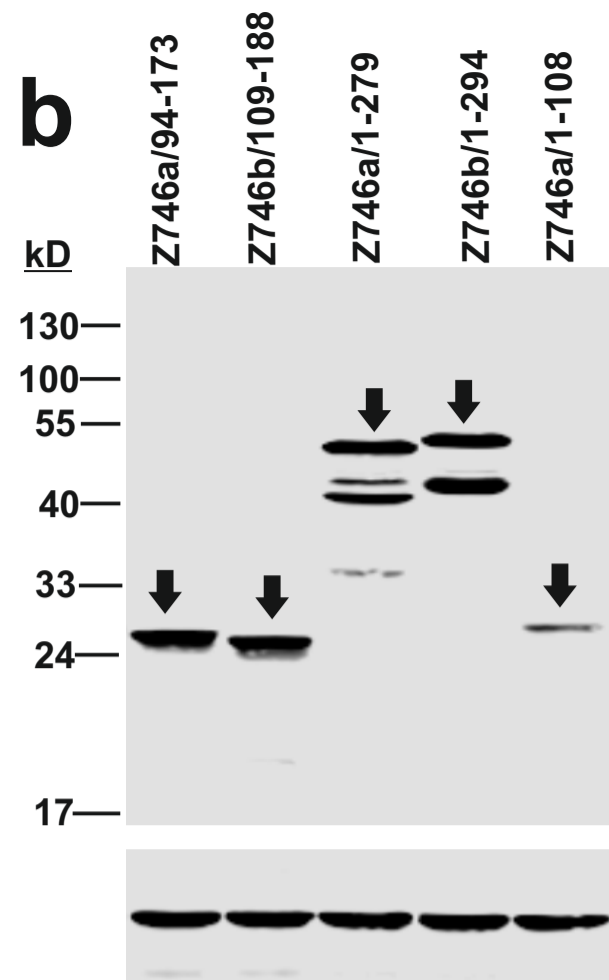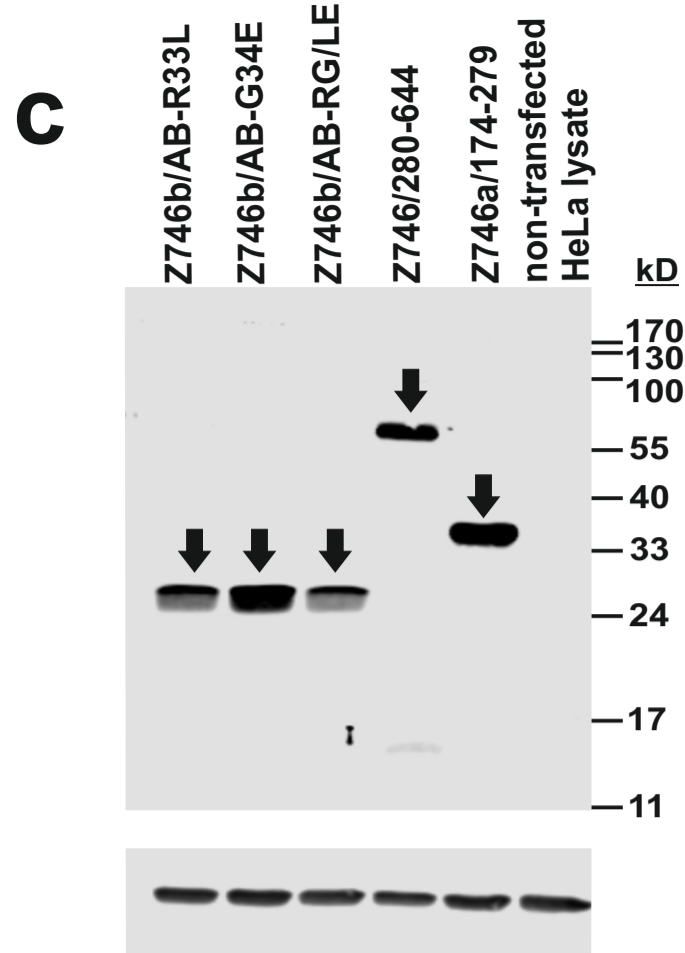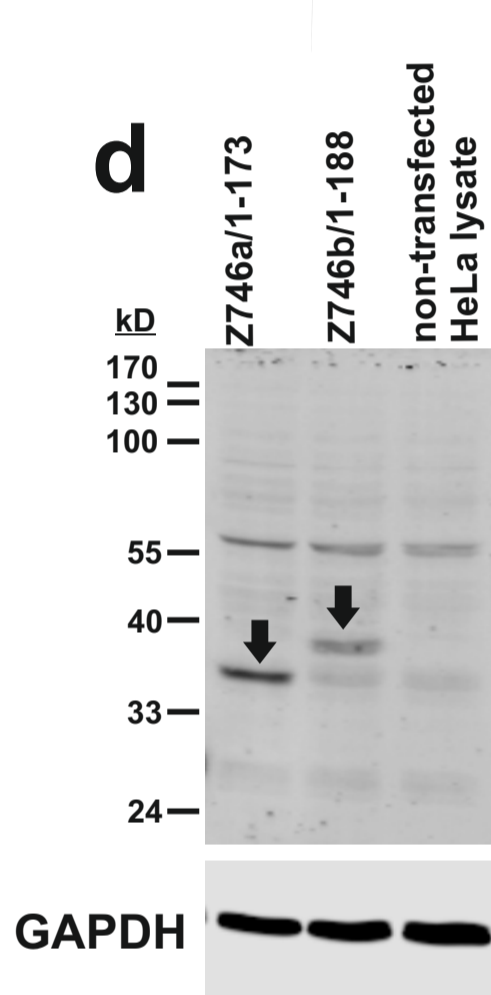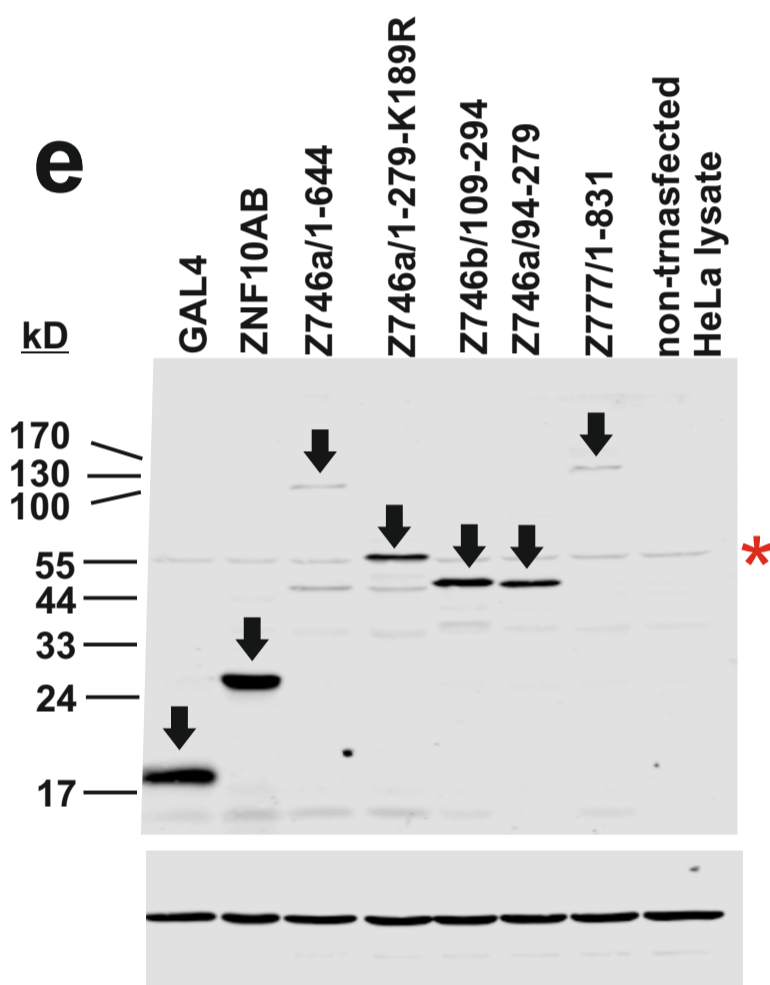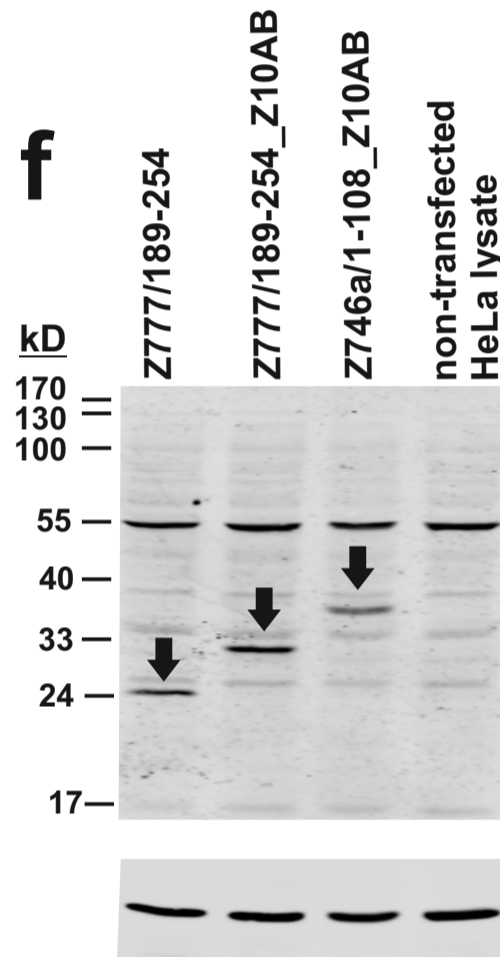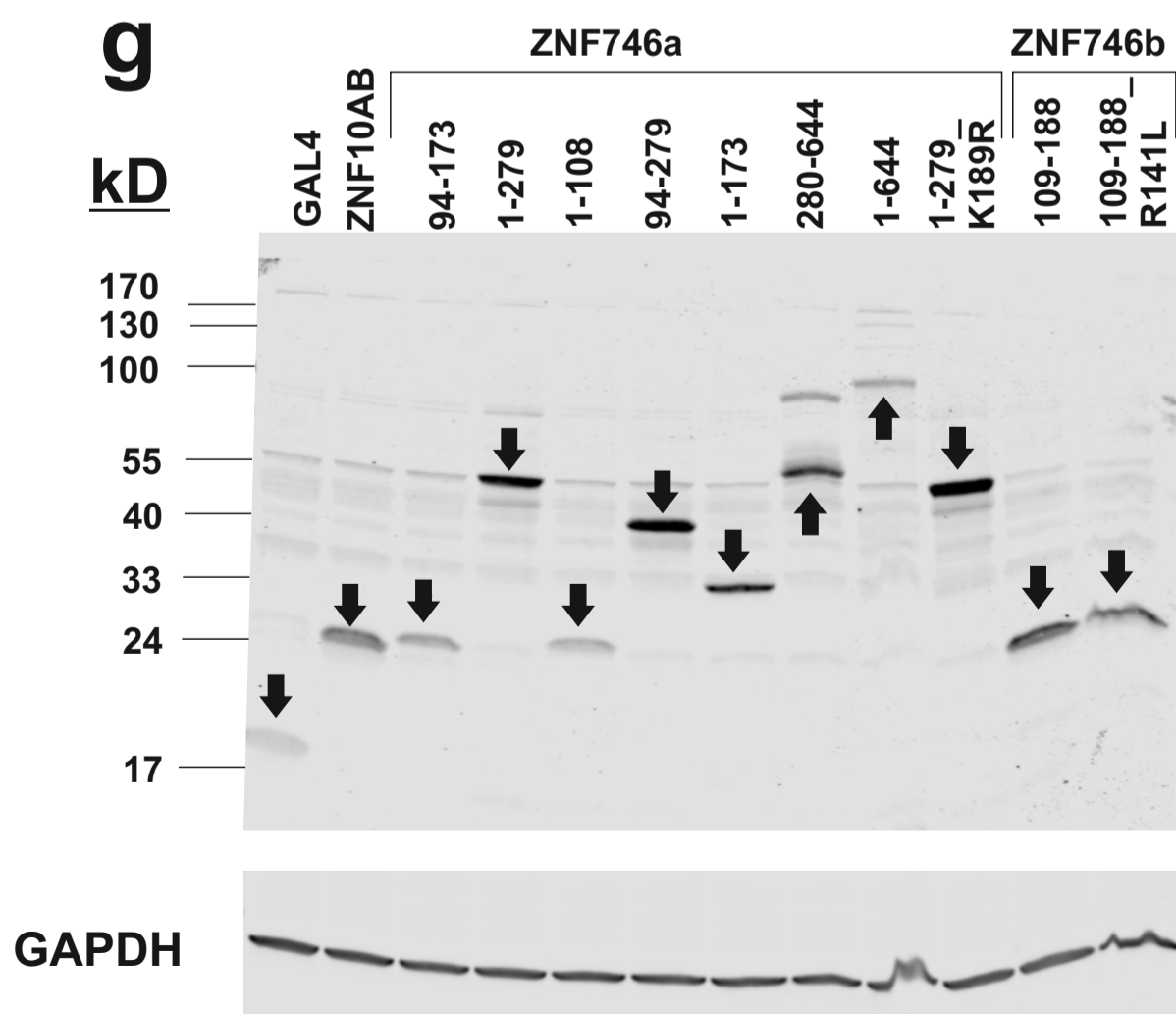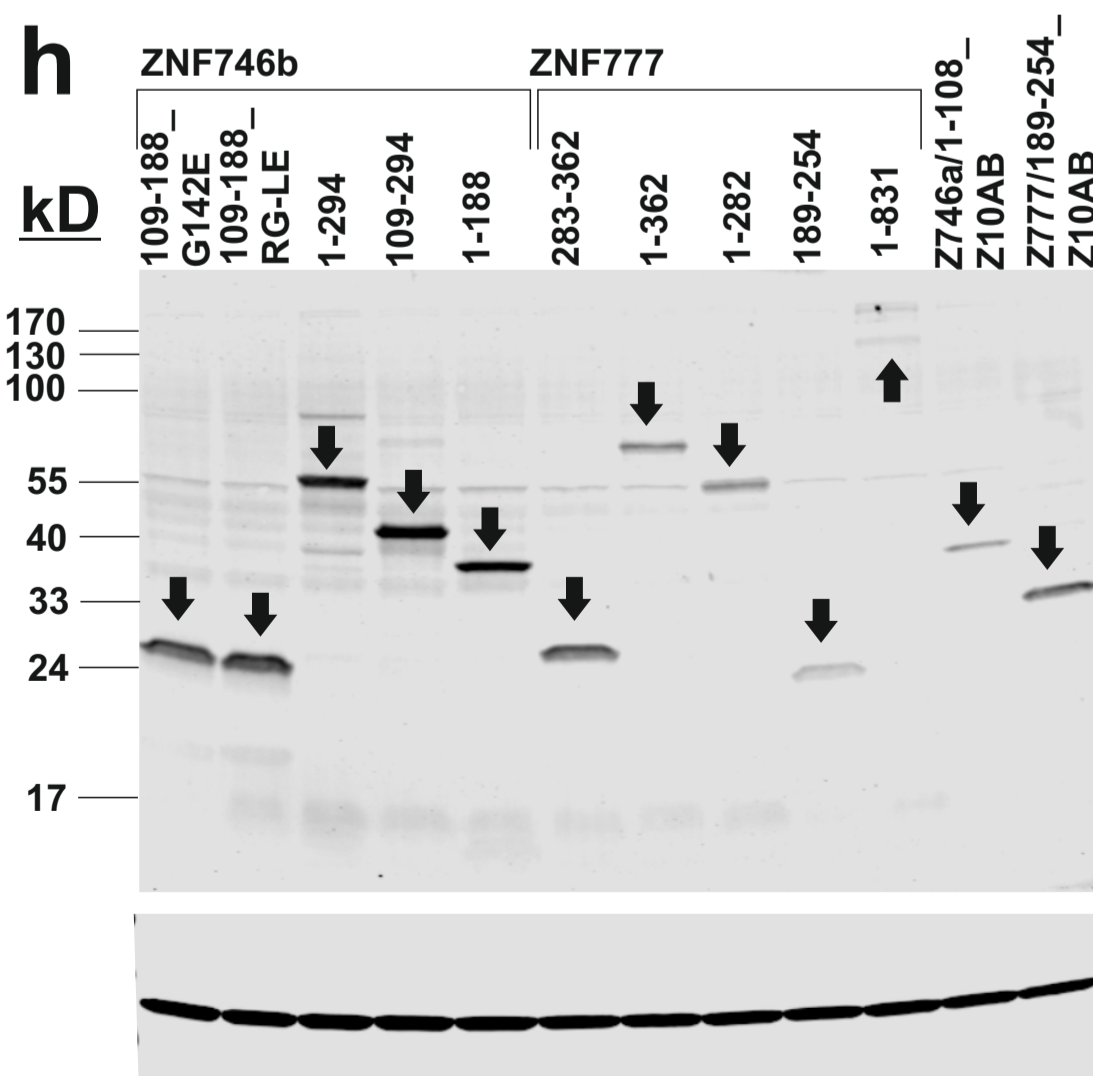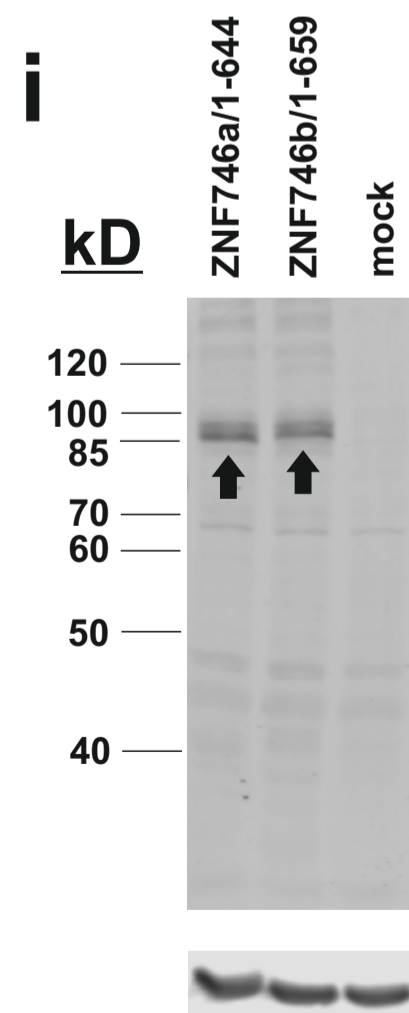

Supplement: Supplementary file 5 — Additional file 5. Verification of expression and expected size of the Gal4 fusion proteins encoded by the constructed pM3 expression vectors using Western blotting in HeLa cells (a, b, c, d, e, f) and HAP1 wild type cells (g - i). Cell extracts made 24 h post-transfection with 1 x SDS sample buffer were probed with rabbit polyclonal antibodies against GAL4 (upper panels; Santa Cruz Biotechnology sc-577 at 0.2 μg/ml) and monoclonal antibodies against endogenous GAPDH (lower panels; Abcam ab8245 at 0.1 μg/ml). Non-transfected HeLa cell lysates are used as negative controls. Black block arrows point to bands of expected size when more than one protein species is visible; * indicates bands due to cross-reactivity of the antibodies. Panels a-i cover all Gal4 fusion protein constructs used in the manuscript. [file 12860_2019_243_MOESM5_ESM.pdf]

**a**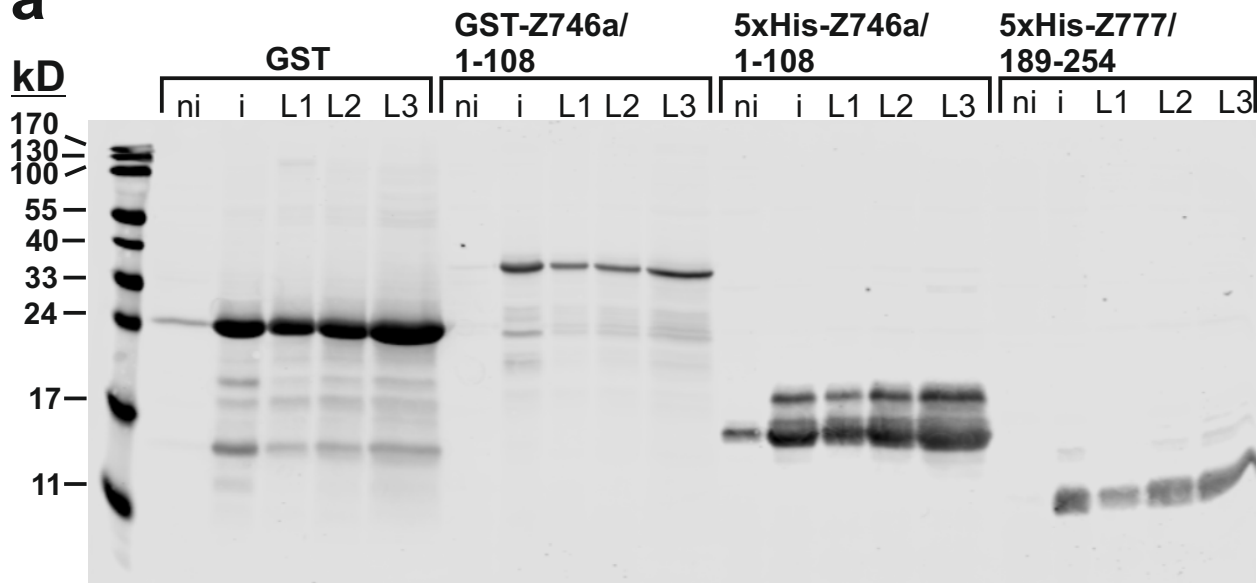**b**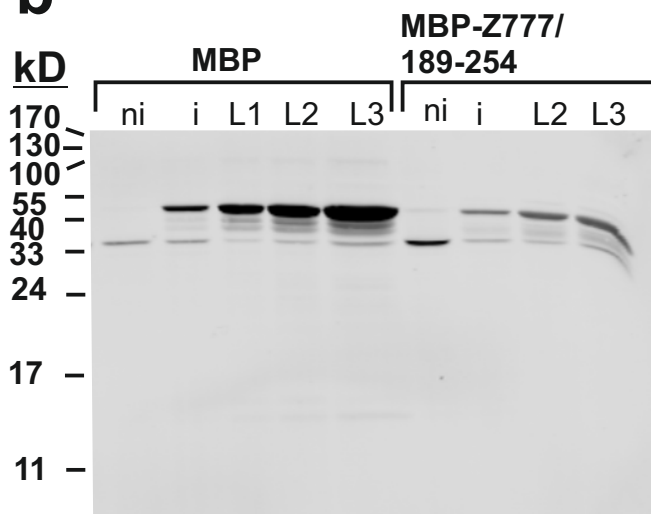**c**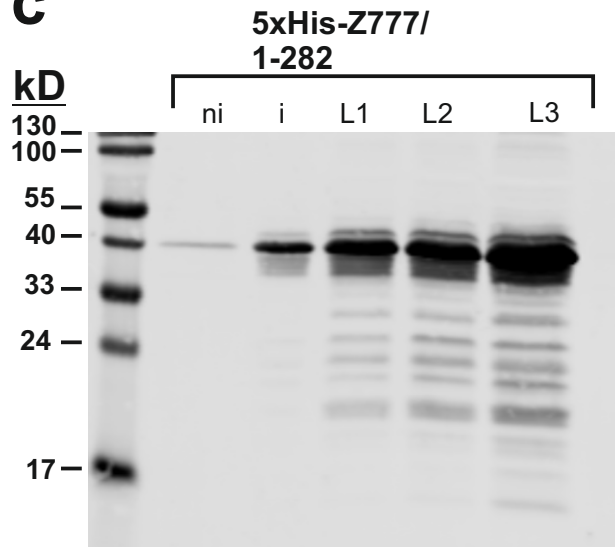

Supplement: Supplementary file 6 — Additional file 6 In vitro expression of DUF3669-containing polypeptides derived from ZNF746 and ZNF777. SoluBL21 (DE3) E. coli were transformed with 10 ng of the indicated prokaryotic constructs. The protein expression was induced by adding 0.1 mM IPTG to the bacterial suspensions at OD600 = 0.4–0.6. Bacterial cell pellets were lysed with 1 x SDS sample buffer (ni = non-induced bacteria, 25 μl total extract; i = bacteria after 4 h induction with IPTG, 25 μl). Soluble crude fractions of recombinant proteins were obtained from induced bacteria that were washed with 1x ice-cooled PBS, re-suspended in lysis buffer and lysed by sonication. Different amounts of bacterial lysates were loaded to the SDS-polyacrylamide gels (L1: 4 μl, L2:8μl, L3:20μl). Extracts were subjected to Western blotting and the membranes probed with polyclonal anti-GST and monoclonal anti-His tag (a, two-color results shown as one black/white overlay representation), polyclonal anti-MBP (b) or monoclonal anti-His tag (c) antibodies. [file 12860_2019_243_MOESM6_ESM.pdf]

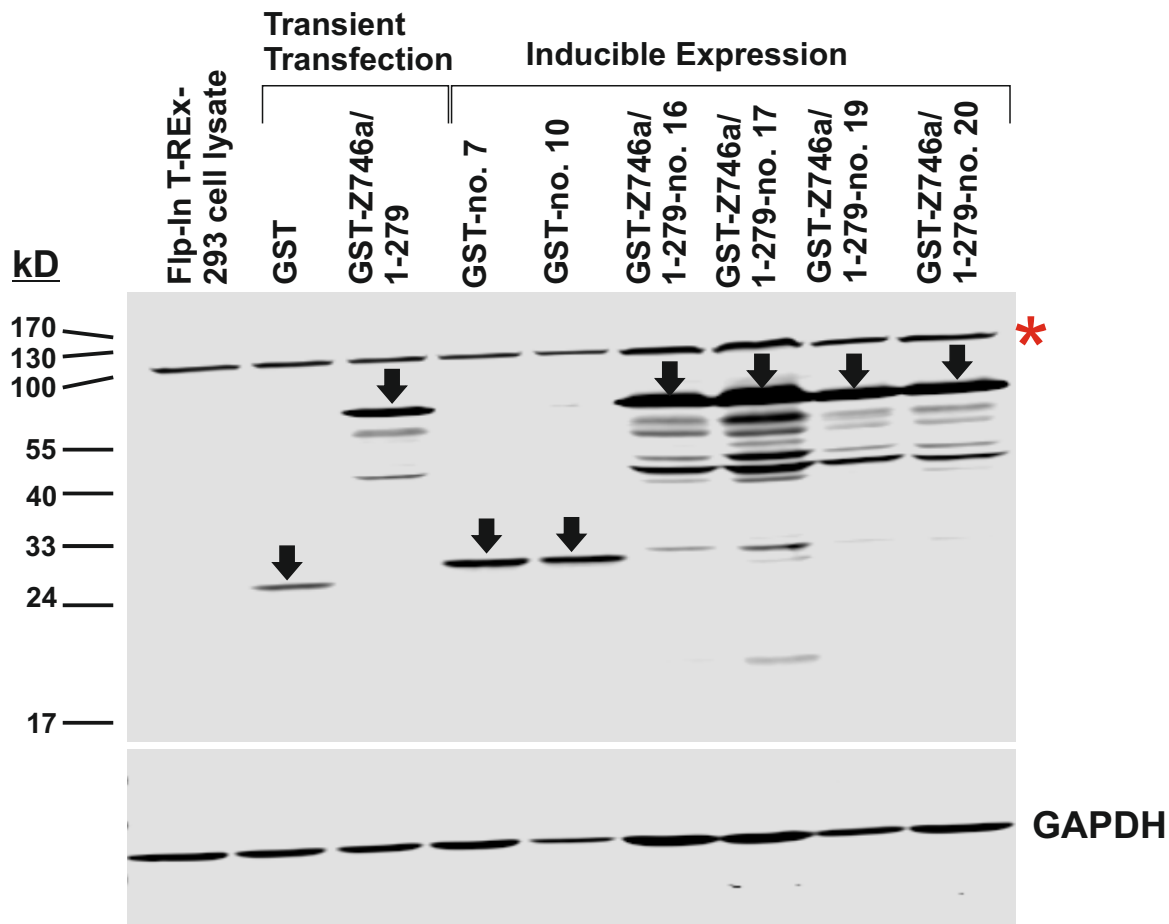

Supplement: Supplementary file 7 — Additional file 7. Analysis of stable HEK293 cell lines expressing GST alone or GST-Z746a/1–279 Western blot analysis of total protein extracts from six clones that survived under the selection of hygromycin B (2 express GST and 4 express GST-Z746a/1–279) alongside extracts from parent cell line Flp-in T-REx-239 cells (negative control) and transiently transfected parent cells expressing GST or GST-Z746a/1–279 (positive controls). Extracts made with 1x SDS sample buffer after a 24-h induction of expression with 2 μg/ml tetracycline. The blot was probed with anti-GST (depicted in the upper part) and anti-GAPDH (lower part) antibodies. Protein bands of the expected size are indicated by arrows. * indicates unspecific bands recognized by the antibodies. [file 12860_2019_243_MOESM7_ESM.pdf]
