# Supplementary material for: DUF3669, a “domain of unknown function” within ZNF746 and ZNF777, oligomerizes and contributes to transcriptional repression
Source: BMC Mol Cell Biol. 2019 Dec 19;20:60. doi: 10.1186/s12860-019-0243-y (PMC6923878; doi:10.1186/s12860-019-0243-y)
Supplement: Supplementary file 4 — Additional file 4. List of PCR primers. [file 12860_2019_243_MOESM4_ESM.pdf]

List of primers used for PCR. Lowercase letters: overhangs that contain restriction sites; uppercase letters: coding sequence. The positions of the oligonucleotides in ZNF746 and ZNF777 exons are indicated relative to the nucleotide position; ZNF746 (isoform a: RefSeq NM_152557.4 and isoform b: NM_001363517.1) and ZNF777 (NM_015694.3).

Name	Position	PCR primers
**Z746a/94-173**	nt 551-790	5′-ccgctcgagtTGGATCCTGCGGCTGCC-3′<br>3′-acgcgtcgactaTGGACTGGGGTCCACAGGAA-5′
**Z746a/1-279**	nt 272-1108	5′-ccgctcgagtATGGCCGAGGCGGTCG-3′<br>3′-acgcgtcgactaTTCCGTGGAGGCTGCTGT-5′
**Z746a/1-173**	nt 272-790	5′-ccgctcgagtATGGCCGAGGCGGTCG-3′<br>3′-acgcgtcgactaTGGACTGGGGTCCACAGGAA-5′
**Z746a/94-279**	nt 551-1108	5′-ccgctcgagtTGGATCCTGCGGCTGCC-3′<br>3′-acgcgtcgactaTTCCGTGGAGGCTGCTGT-5′
**Z746a**	nt 272-2206	5′-ccgctcgagtATGGCCGAGGCGGTCG-3′<br>3′-acgcgtcgacTCACATGTCCCCGCCAT-5′
**Z746a/174-279**	nt 791-1108	5′-ccgctcgagtGGCTCGGGGCC-3′<br>3′-acgcgtcgactaTTCCGTGGAGGCTGCTGT-5′
**Z746a/280-644**	nt 1109-2206	5′-ccgctcgagtATGgATGTAAAAATTGTAATAAAAACAGA-3′<br>3′-cgcgtcgacTCACATGTCCCCGCCAT-5′
**Z746a/1-108**	nt 272-595	5′-ccgctcgagtATGGCCGAGGCGGTCG-3′<br>3′-acgcgtcgactaCTTAGGGGACTCCCCCTTGC-5′
**Z746b/109-188**	nt 598-837	5′- ccgctcgagtGTGCCCGTGACCTTTGATGATG -3′<br>3′-acgcgtcgactaTGGACTGGGGTCCACAGGAA-5′
**Z746b/1-294**	nt 274-1155	5′-ccgctcgagtATGGCCGAGGCGGTCG-3′<br>3′-acgcgtcgactaTTCCGTGGAGGCTGCTGT-5′
**Z746b/1-188**	nt 274-837	5′-ccgctcgagtATGGCCGAGGCGGTCG-3′<br>3′-acgcgtcgactaTGGACTGGGGTCCACAGGAA-5′
**Z746b/109-294**	nt 598-1155	5′- ccgctcgagtGTGCCCGTGACCTTTGATGATG -3′<br>3′-acgcgtcgactaTTCCGTGGAGGCTGCTGT-5′
**Z777/283-362**	nt 1110-1350	5′-ccgctcgagtGTCCCTGTCACATTTGATGATGT-3′<br>3′-acgcgtcgactaAGCACTGGGATCTGTCGG-5′
**Z777/1-362**	nt 264-1350	5′-ccgctcgagtATGGAGAACCAACGCTCATC-3′<br>3′-acgcgtcgactaAGCACTGGGATCTGTCGG-5′
**Z777/1-282**	nt 264-1109	5′-ccgctcgagtATGGAGAACCAACGCTCATC-3′<br>3′-acgcgtcgactaCTTGGGAACTTCTCCATTGCT-5′
**Z777/189-254**	nt 828-1025	5′-ccgctcgagtTGGGCTGCCGTCCAA-3′<br>3′-acgcgtcgactaCCGCCTCTGCAGCAGC-5′
